# Supplementary material for: MAVEN2: An Updated Open-Source Mass Spectrometry Exploration Platform
Source: Metabolites. 2022 Jul 25;12(8):684. doi: 10.3390/metabo12080684 (PMC9330773; doi:10.3390/metabo12080684)
Supplement: Supplementary file 1 [file metabolites-12-00684-s001.zip › metabolites-1827771_proof-supplementary/Supplementary_Information.pdf]

Dataset S1: Lipidomics Analysis

See associated file “Dataset_S1_Lipidomics_Analysis.zip”

Figure S1: Retention Balance Point Methodology

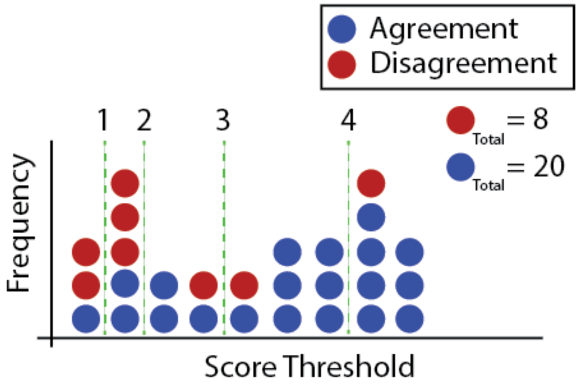

1. R = (19/20)-(5/8) = 0.325
2. R = (17/20)-(3/8) = 0.475 (RBP)
3. R = (14/20)-(2/8) = 0.45
4. R = (7/20)-(1/8) = 0.225

As a surrogate for gold standards or labeled data, we used the annotation agreement between CalicoLipids and MS-DIAL spectral libraries to estimate library-specific score thresholds which could be applied broadly to all annotated features. The use of agreement between multiple identification approaches has been used extensively in the past as a metric for performance in mass spectrometry datasets, for example, when comparing peptide spectrum matches identified by different proteomics search engines and metabolite annotations produced by different metabolomics identification algorithms. An “agreement” indicates that both the CalicoLipids and MS-DIAL spectral libraries returned the same annotation for a given feature, while a “disagreement” indicates that the two libraries annotated the feature differently. For this analysis, an “agreement” requires that both features are assigned the same lipid class, adduct form, and acyl chain lengths. A “disagreement” must differ in all of these ways. MS-DIAL and CalicoLipids characterized lipid classes slightly differently, which necessitated the development of some alignment functions, as well as careful manual review when aligning features. Fortunately, the MAVEN peak detection and grouping step is deterministic, so the unannotated set of peak groups was identical in all searches carried out. A complete analysis of this work, including all necessary raw files and functions, is available in **Dataset S1**.

Annotation agreement likely indicates that both libraries suggest the correct annotation, with the alternative being that both have independently suggested the same wrong annotation. Annotation disagreement requires that at least one annotation is incorrect, though it is possible that both annotations could be incorrect. We assumed that

annotation agreement is generally a favorable attribute, and annotation disagreement is generally an unfavorable attribute. We found that for both the CalicoLipids and MS-DIAL libraries, annotation agreement is generally associated with higher score values, while annotation disagreement is generally associated with lower score values, though the score distributions are overlapping **(Figure S3)** Review of the spectral contents of the CalicoLipids and MS-DIAL libraries reveals that the CalicoLipids spectral library contains more fragments per library entry than the MS-DIAL spectral library (CalicoLipids averages 11.0 fragments per compound ion, while MS-DIAL averages 6.7 fragments per compound ion, see **Dataset S1**). This yields systematically higher hypergeometric score values for CalicoLipids library matches compared to MS-DIAL matches, as hypergeometric scores increase as the absolute number of fragment matches increases. With more fragments available to match, more fragment matches should be discovered.

For each library, we determined the proportion of annotation agreements and disagreements retained after removing score values below the threshold (the retention fraction difference, or RFD). We repeated this procedure for all reasonable score threshold values, recording the RFD computed at each hypergeometric score threshold value to create distributions of library-specific retention difference values. After smoothing the library-specific retention difference distributions, we observed similarity between the shape of the two distributions, including the presence of a prominent global maximum in each distribution, which we termed the Retention Balance Point (or RBP), and used as the corresponding library's score cutoff value. This maximum of each distribution is the value that maximizes the difference between the proportion of agreements and the proportion of disagreements retained at any score threshold value.

Our interpretation of the distribution maxima is as follows: our procedure is analogous to balancing sensitivity and specificity, where the retention of annotation agreements is analogous to sensitivity (retaining true positives), and the exclusion of annotation disagreements is analogous to specificity (excluding true negatives). Critically, annotation disagreements may be fully incorrect (both annotations are wrong) or half-incorrect (only one annotation is wrong), so we may not translate agreement and disagreement into true positives and true negatives without further assumptions. For this reason, without additional assumptions, we are unable to render a false discovery rate (FDR) using our approach. However, suppose we make the assumption that in general, higher scores are more likely to be real identifications. This seems reasonable for the hypergeometric score test, as higher scores correspond to a higher number of fragment matches. Assuming that the libraries are built with informative, meaningful fragments, more fragment matches ought to indicate a higher likelihood of a correct annotation. Features that are differently annotated by each spectral library (the disagreements) should follow the same trend, in reverse (e.g., a failure to match fragments indicates a lower likelihood of correct annotation). While we cannot explicitly segment our disagreements into correct and incorrect annotations, as the score threshold increases, we suggest that the ratio of correct annotations to incorrect annotations should increase. The maximum value of an agreement and disagreement retention difference distribution can be understood as the point at which a change in character of the disagreements occurs, specifically, where the proportion of half-correct annotations to doubly-incorrect annotations is maximized. It's worth mentioning that the retention plots for one spectral

library may be transformed into the retention plot of the other spectral library by simple shrinking/stretching mathematical transformations. Understood this way, we can view our library-specific thresholds not as arbitrary cutoffs, but as a fundamental property of each library's agreement and disagreement retention fraction difference distribution. We may use a library-specific hypergeometric score threshold as a surrogate for a probability or FDR value, above which we would designate matches as "real", and below which we would discard as spurious.

In the above figure, the approach of computing the retention balance point (RBP) is shown pictorially: Score thresholds are drawn across the entire distribution of scored matches (green dotted lines). At each score threshold, the proportion of agreements at or above the threshold and proportion of disagreements at or above the threshold is recorded. In the figure, this is computed at 4 different values, shown as "R", with the RBP for this example corresponding to data point #2. The total number of agreements and disagreements, 20 and 8 respectively, is shown in the figure.

Figure S2: Agreement and Disagreement Score Distributions and Retention Balance Points (A)

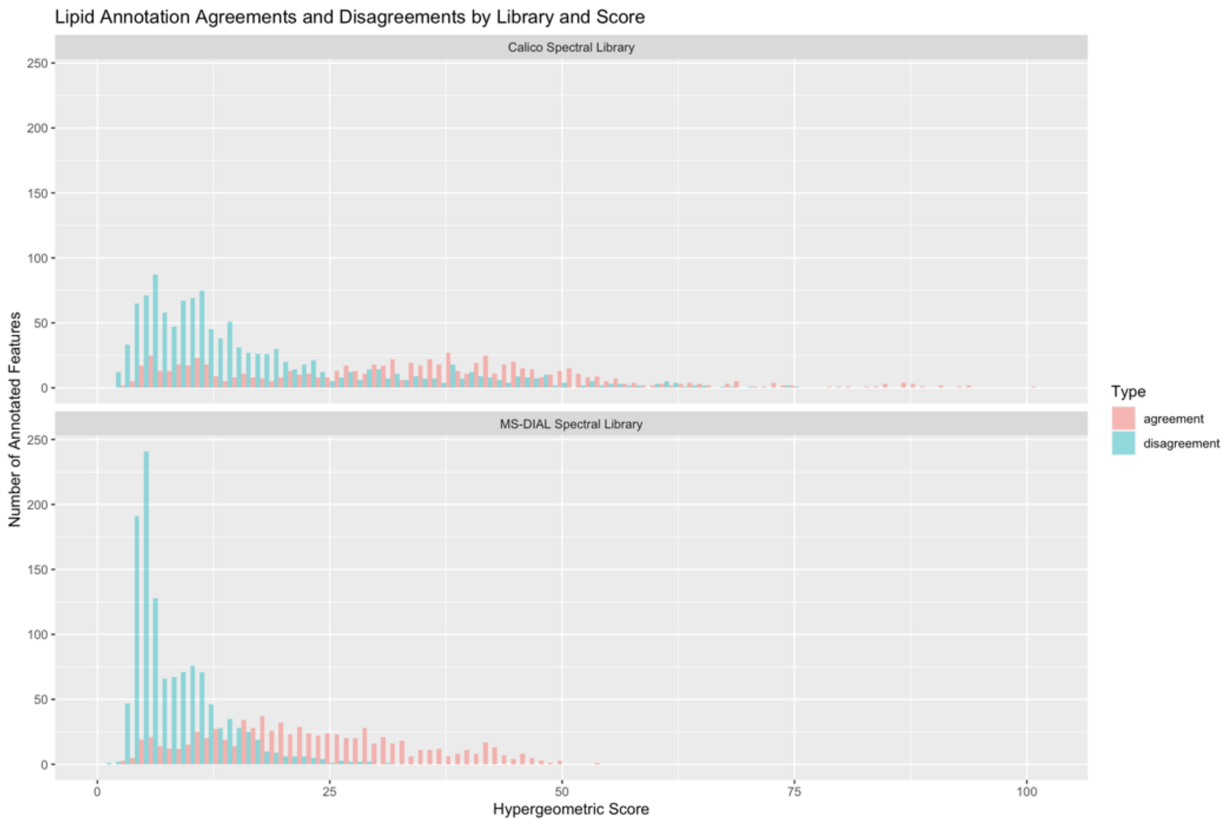

(B)

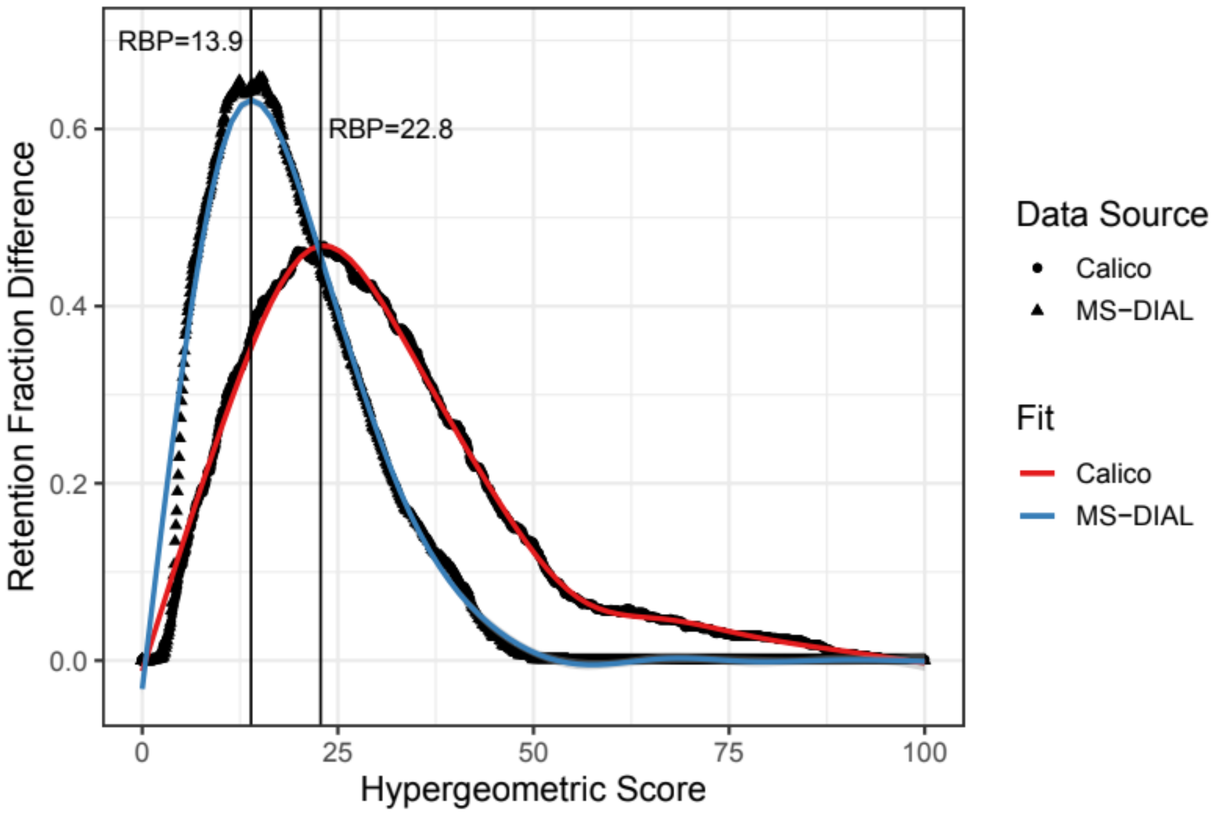

(A) Agreements and disagreements count histograms, segregated by library. Inspection of counts of features annotated by both the CalicoLipids (top) and MS-DIAL (bottom) spectral libraries indicates that features annotated the same way by both libraries (agreements) and features annotated differently between libraries (disagreements) form distinct hypergeometric score distributions. This suggested the existence of a score threshold that optimally separated these distributions, which motivated the development of the retention balance point (RBP) approach. **(B)** Overlying the distributions of retention fraction difference values computed at different hypergeometric score thresholds is shown overlaid with fitted curves (CalicoLipids raw data shown as circles, fit shown as red line, MS-DIAL raw data shown as triangles, fit shown as blue line). The distributions have a similar unimodal shape with a single local maximum (the retention balance point, or RBP). In this dataset, the RBP was found to exist at a hypergeometric score threshold of 13.9 for the MS-DIAL spectral library, and 22.8 for the CalicoLipids spectral library.

Table S1: Lipid Fragmentation Standards

Class	Class Abbreviation	Standards	Other sources and Citations	precursors fragmented
**Bisdiacylglycerolphosphate**	BDP	BDP(18:1/18:1/18:1/18:1)		[M-H]-, [M+H]+, [M+Na]+
**Bismonoacylglycerolphosphate**	BMP	BMP(14:0/14:0), BMP(18:1,18:1)		[M-H]-, [M+H]+, [M+Na]+
**Cardiolipin**	CL	CL(16:0/18:1/16:0/18:1), CL(18:1/18:1/18:1/18:1)		[M-H]-, [M-2H]2-

**Carnitine**	Carn	Carn(16:0), Carn(12:0)		[M+H]+
**CDP-diacylglycerol**	CDP-DG	CDP-DG(16:0/16:0), CDP-DG(18:1/18:1)		[M-H]-, [M+H]+, [M+Na]+
**Ceramide**	Cer	Cer(d18:0/12:0), Cer(d18:1/m18:0), Cer(d18:1/24:0), Cer(t18:0/18:0), Cer(t18:0/24:0)		[M-H]-, [M+H]+, [M+Na]+
**Ceramide Phosphate**	Cer-P	Cer-P(d18:1/12:0), Cer-P(d18:1/24:0)		[M-H]-
**Ceramide Phosphatidylethanolamine**	CPE	CPE(d17:1, 12:0), CPE(d18:1,24:1), CPE(d18:1,24:0)		[M-H]-, [M+H]+, [M+Na]+
**Ceramide Phosphatidylinositol**	CPI		by analogy to CPE, X. Han 2017	
**Cholesterol Ester**	CE	CE(17:0)		[M+Na]+
**diacylglycerol**	DG	DG(14:0/14:0), DG(16:0/16:0), DG(18:0,18:0), DG(16:0,18:1)		[M+Na]+
**digalactosyldiacylglycerol**	DGDG		X. Han 2017	
**Diacylglyceryltrimethylhomo-Ser**	DGTS	DGTS(16:0,16:0)		[M+H]+
**dimethyl-phosphatidylethanolamine**	DMPE		by analogy to PE, spectra from complex sample	
**Ergosterol ester**	ErgE		by anaolgy to CE	
**Disialoganglioside GD1a**	AcGD1a	GD1a (d18:1/18:0)		[M+H]+, [M+2H]2+, [M+Na]+, [M-H]-, [M-2H]2-
**Disialoganglioside GD1b**	AcGD1b	GD1b (d18:1/18:0)		[M+H]+, [M+2H]2+, [M+Na]+, [M-H]-, [M-2H]2-
**Disialoganglioside GD2**	AcGD2	GD2 (d18:1/18:0)		[M+H]+, [M+Na]+, [M-H]-, [M-2H]2-
**Disialoganglioside GD3**	AcGD3	GD3 (d18:1/18:0)		[M+H]+, [M+Na]+, [M-H]-, [M-2H]2-
**ether lysophosphatidylcholine**	LPC	LPC(p18:0)		[M-H]-, [M+H]+, [M+Na]+
**ether lysophosphatidylethanolamine**	LPE	LPE(p18:0)		[M-H]-, [M+H]+, [M+Na]+
**fatty acid**	FA		X. Han 2017	
**FAHFA**	FAHFA	5-PAHSA, 12-PAHSA		[M-H]-, [M+Na]+
**Globotriaosylceramide**	GB3		X. Han 2017	
**Neu5Gc Monosialoganglioside GM2**	GcGM2		by anaology to AcGM2	
**Neu5Gc Monosialoganglioside GM3**	GcGM3		by analogy to AcGM3	
**Hemi-bismonoacylglycerolphosphate**	HemiBMP	HemiBMP(18:1,18:1,18:1)		[M-H]-
**Galactosyl-Ceramide + Glucosyl Ceramide**	Hex-Cer	Galactosyl-Ceramide(d18:1/16:0), Glucosyl-Ceramide(d18:1,16:0), Glucosyl-Ceramide(d18:1,17:0)		[M-H]-, [M+H]+, [M+Na]+
**Lactosyl Ceramide**	LacCer	LacCer(d18:1,17:0), Glucosyl-Ceramide(d18:1,24:1)		[M-H]-, [M+H]+, [M+Na]+

**lyso Ceramide Phosphatidylethanolamine**	LysoCPE	LysoCPE(d18:1)		[M-H]-, [M+H]+, [M+Na]+
**lyso Ceramide Phosphatidylinositol**	LysoCPI	LysoCPI(d18:1)		[M-H]-, [M+H]+, [M+Na]+
**lyso sphingomyelin**	LysoSM	LysoSM(d17:1), LysoSM(d18:1)		[M+H]+, [M+Na]+
**glucosyl sphingosine, galactosyl sphingosine (pyschosine)**	LysoHexCer		X. Han 2017	
**Lysophosphatidic acid**	LPA	LPA(16:0), LPA(18:1)		[M-H]-
**Lysophosphatidyl glycerol**	LPG	LPG(16:0), LPG(18:1)		[M-H]-, [M+H]+, [M+Na]+
**Lysophosphatidyl inositol**	LPI	LPI(16:0), LPI(18:1)		[M-H]-, [M+H]+, [M+Na]+
**Lysophosphatidylcholine**	LPC	LPC(18:0/0:0), LPC(15:0,0:0), LPC(16:0,0:0), LPC(18:1,0:0)		[M-H]-, [M+H]+, [M+Na]+
**Lysophosphatidylethanolamine**	LPE	LPE(18:0)		[M-H]-, [M+H]+, [M+Na]+
**lysophosphatidylserine**	LPS	LPS(16:0)		[M-H]-, [M+H]+, [M+Na]+
**Monoacylglycerol**	MG	MG(12:0), MG(18:0)		[M-H]-, [M+H]+, [M+Na]+
**Monogalactosyldiacylglycerol**	MGDG	MGDG(18:0,18:0)	X. Han 2017	[M-H]-, [M+H]+, [M+Na]+
**mannose-(inositol phosphate)2-ceramide**	MIP2C		spectra from complex sample	[M-H]-
**mannose-inositol phosphate-ceramide**	MIPC		spectra from complex sample	[M-H]-
**mono-methyl phosphatidylethanolamine**	MMPE		by analogy to PE, spectra from complex sample	
**N-Acylphosphatidylethanolamine**	N-acyl-PE		X. Han 2017	
**N-Acylphosphatidylserine**	N-acyl-PS		X. Han 2017	
**monoether phosphatidylcholine**	Alkyl_PC	PC(p18:0/22:6), PC(p18:0/18:1), PC(p18:0/20:4)		[M+FA-H]-, [M+H]+, [M+Na]+
**monoether phosphatidylethanolamine**	Alkyl_PE	PE(p18:0,18:1), PE(p18:0,24:0)		[M-H]-, [M+H]+, [M+Na]+
**Monosialoganglioside GM1**	AcGM1	GM1 (d18:1/18:0)		[M-H]-, [M+H]+
**Monosialoganglioside GM2**	AcGM2	GM2 (d18:1/18:0)		[M-H]-, [M+H]+
**Monosialoganglioside GM3**	AcGM3	GM3 (d18:1/18:0)		[M-H]-, [M+H]+
**N-acyl-ethanolamine**	Ethanolamine		X. Han 2017	
**N-acyl Taurine**	Taurine	Taurine(18:0)		[M-H]-, [M+H]+
**Phosphatidic acid**	PA	PA(18:1/18:1), PA(16:0/18:1), PA(18:0/18:1)		[M-H]-
**Phosphatidylcholine**	PC	PC(16:0/18:1), PC(16:0/18:0), PC(18:0/20:4)		[M+FA-H]-, [M+H]+, [M+Na]+
**Phosphatidylethanolamine**	PE	PE(16:0/18:1), PE(16:0/20:4)		[M-H]-, [M+H]+, [M+Na]+
**phosphatidylglycerol**	PG	PG(16:0/18:1), PG(18:0,18:0)		[M-H]-, [M+H]+, [M+Na]+
**Phosphatidylinositol**	PI	PI(16:0/18:1), PI(18:0,20:4)		[M-H]-, [M+H]+, [M+Na]+

**Phosphatidylserine**	PS	PS(16:0/16:0), PS(16:0/18:1)		[M-H]-, [M+H]+, [M+Na]+
**Sphingomyelin**	SM	SM(d18:1/18:0), SM(d18:1,24:0), SM(d18:1/18:1)		[M-H]-, [M+H]+, [M+Na]+
**sphingosine phosphate**	LCB-P	LCB-P(d20:1)		[M-H]-, [M+H]+
**Sphingosine/Sphiganine**	LCB	LCB(d18:1), LCB(d18:0), LCB(d20:0)		[M+H]+
**Sulfatide**	Sulfatide	Sulfatide(d18:1/17:0), Sulfatide(d18:1/m18:0), Sulfatide(d18:1/12:0), Sulfatide(d18:1/24:1)		[M-H]-
**Tetrasialoganglioside GQ1b (NH4+salt)**	AcGQ1b	GQ1b (d18:1/18:0)		[M+H]+, [M+2H+]2+, [M-2H]2-, [M-3H]3-
**triacylglycerol**	TG	TG(12:0/12:0/12:0), TG(14:0/14:0/14:0), TG(16:0/16:0/16:0), TG(18:0/18:0/18:0)		[M+Na]+
**Trisialoganglioside GT1b (NH4+salt)**	AcGT1b	GT1b (d18:1/18:0)		[M+H]+, [M+2H+]2+, [M+Na]+, [M-2H]2-

Dataset S2:

See associated file “Dataset_S2_Metabolomics_Library_Files.zip”

Dataset S3:

See associated file “Dataset_S3_Metabolomics_Example_Analysis.zip”

Figure S3: Gold Standards Metabolomics Analysis

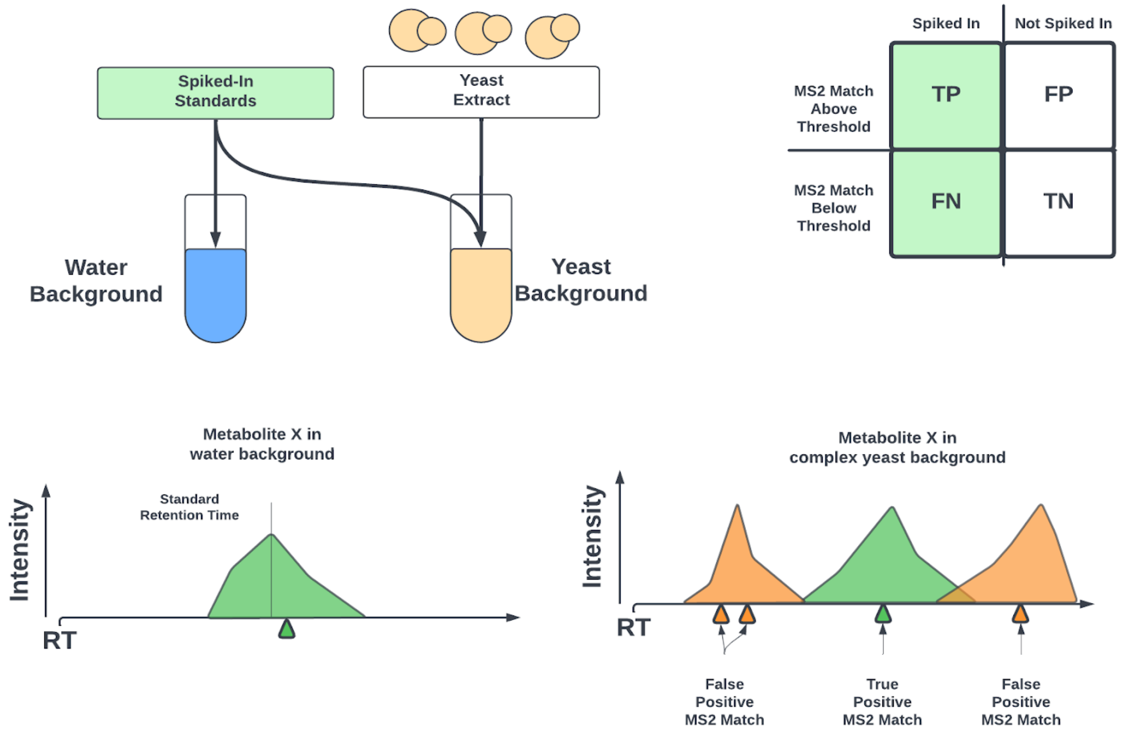

Samples were generated as described in **Methods**. A series of purified chemical standards were purchased and spiked into a subset of samples. Each chemical standard spiked into samples was spiked into exactly 2 of the 10 samples associated with each search. We retrieved a list indicating which two samples contained a particular chemical standard. In general, samples contained many chemical standards, though the number of chemical standards per sample was constrained to 37 metabolites.

MAVEN2 was launched, and a series of searches were undertaken using different samples and spectral libraries. In total, four searches were performed, using the following combinations of samples and libraries: (1) negative library, standards spiked into a water background; (2) positive library, standards spiked into a water background; (3) negative library, standards spiked into a yeast background; and (4) positive library, standards spiked into a yeast background. There were 10 samples and 4 blanks included in each case. The spectral libraries used are those described in **Methods**, and available in **Dataset S2**. For each search, a Peaks search was carried out using all default Peak Detection parameters, and default Peak Scoring parameters except for the minimum number of fragments was set to 0, the minimum score was set to 0, and we required that a compound's associated adduct matched the searched adduct match for compound matches. All searches were saved as .mzrollDB files.

The data from MAVEN searches and standard - sample mapping data were imported into an R script (available in **Dataset S4**). For searches associated with water samples, annotation correctness was determined based on the quantitative profile of identified compounds. Specifically, a peak group - compound match was only considered correct if peak intensity was observed in both spike-in samples, and the median peak intensity of this intensity was greater or equal to twice the median intensity of the peak intensity of all of the other samples in the search (if data was not present in a sample, the intensity was set to the limit of detection of 4096 arbitrary intensity units). Once this process was carried out over the search set, we obtained a list of compounds that were correctly identified. Any compounds that could not be correctly identified were excluded from further analysis. The retention times associated with correctly identified peak groups were noted. We used the set of all accurately identified compounds from the water samples to generate a table of compound retention times, which we used to assess correctness among the yeast background samples. Only compounds where a correct identification could be found in the water samples evaluated in the yeast background samples.

Using the list of compounds that were accurately identified in the search set, we explored accuracy, precision, and recall as a function of MS2 score threshold, using a variety of scoring types (dot product score, fraction of library fragments matched, hypergeometric score, MVH score, number of matched library fragments, proportion of the library spectrum TIC matched). Peak groups were considered identifications only if their matched MS2 score was above the threshold. The true identity of each peak group was revealed by the standard spike-ins, and data from both positive and negative modes were combined.

Peak groups with a hypergeometric score below the hypergeometric score threshold were excluded from further consideration. Different scoring methods have different ranges - for instance, the cosine score ranges from 0 to 1, while the hypergeometric score is unbounded. To compare different scoring approaches to each other, we normalized all scores by the maximum observed score of any identification. This allowed us to consider scoring thresholds of every scoring type as a fraction of the maximum observed score.

For both water and yeast background samples, positive and negative modes were combined to produce the response curves shown in **(Figure 3)**. Response curves were generated by iterating through results and excluding entries that had a hypergeometric score below the hypergeometric score threshold. True positive rate (TPR) was always calculated as precision = TP / (TP + FP) (true positive rate equals number of true positives divided by the sum of true positives and false positives). An R markdown analysis script, mzrolldb files, standards list, and screenshots are available in **Dataset S4**.

Figure S4: Metabolite Scoring Performance

(A) Precision of various scoring algorithms in identification of gold standard spike-ins applied to water and yeast backgrounds.

Precision is higher in the water background due to less interference from background metabolites. Percent of TIC matched had the worst performance, and MVH/Hypergeometric had the best performance.

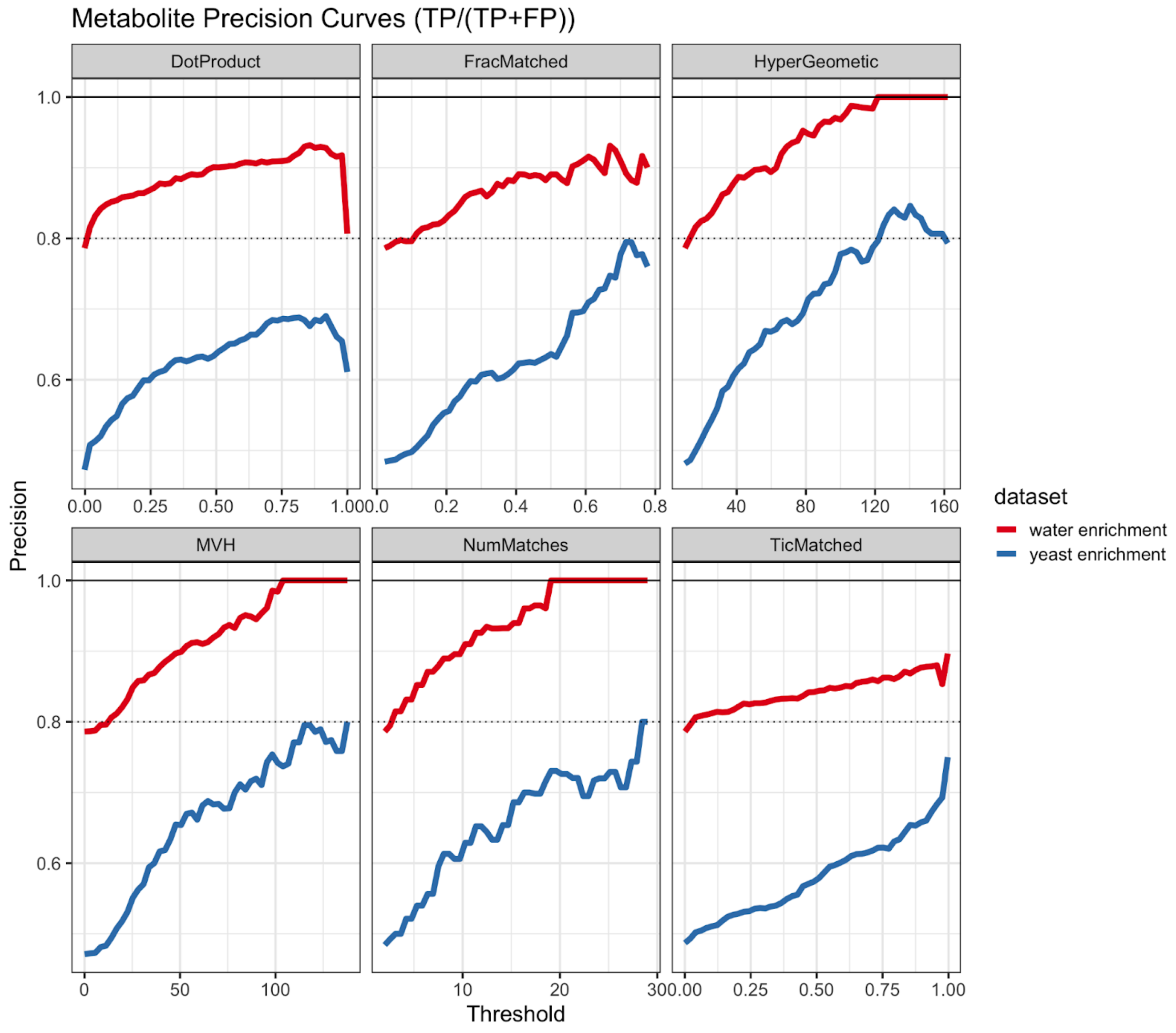

(B) Recall of various scoring algorithms in identification of gold standard spike-ins applied to water and yeast backgrounds. Percent TIC matched had overall best recall however at the cost of lower precision. Of these six scoring types, only TIC matched and DotProduct considered spectral intensity values. These scoring types had similar recall response curves.

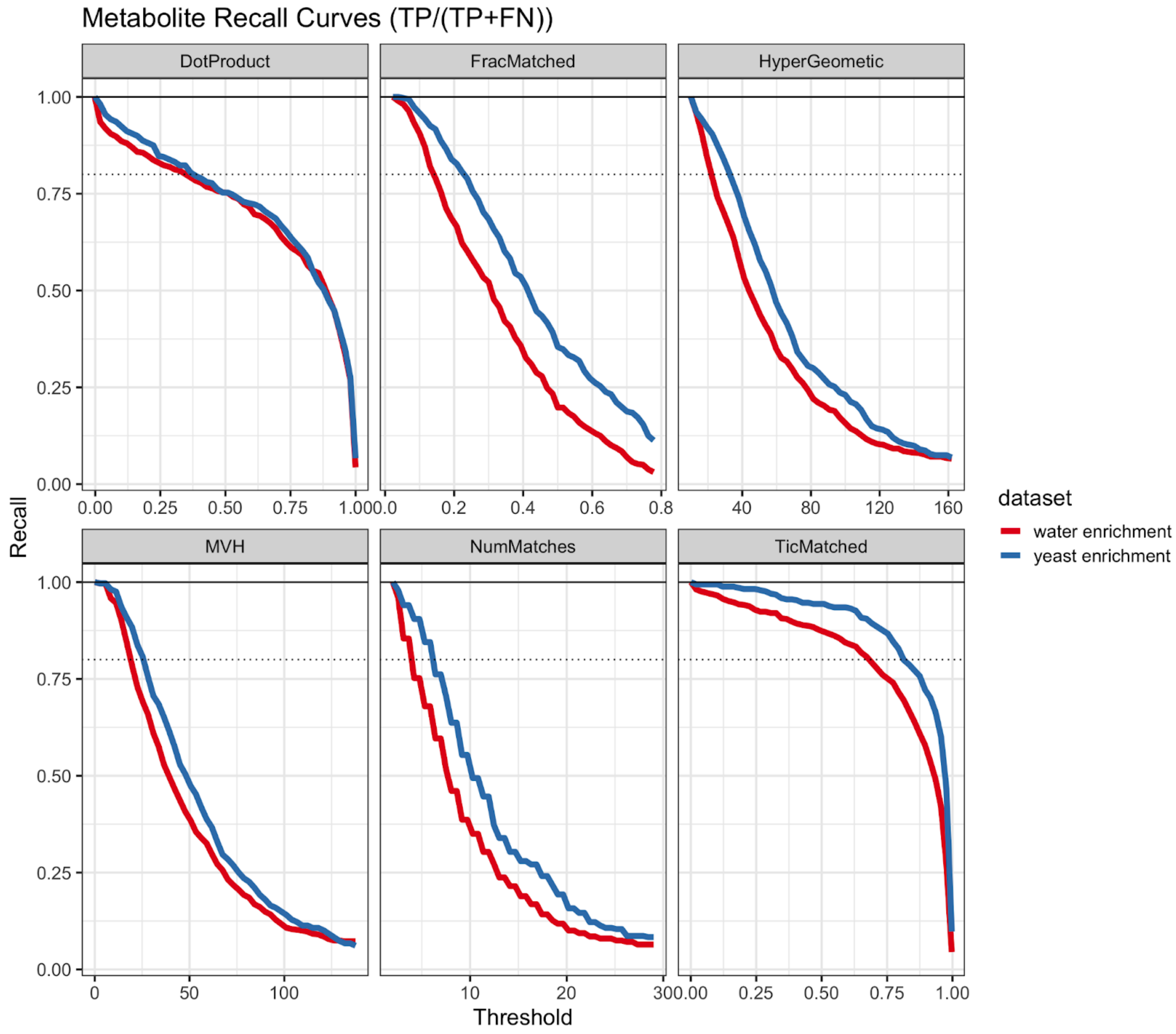

Table S2: Metabolite IDs in Water Background

	Score Threshold (%Max Value)				
**MS2 SCORE**	>0%	25%	50%	75%	100%
**WATER BACKGROUND TRUE POSITIVES**					
**DotProduct**	577	475	434	352	25
**FracMatched**	577	384	207	81	18
**HyperGeometic**	577	307	127	59	38
**MVH**	577	305	134	60	42
**NumMatches**	577	298	124	54	37
**TicMatched**	577	533	503	433	26
**WATER BACKGROUND FALSE POSITIVES**					
**DotProduct**	157	70	48	35	6
**FracMatched**	157	77	28	8	2
**HyperGeometic**	157	39	7	0	0
**MVH**	157	46	11	0	0
**NumMatches**	157	41	9	0	0
**TicMatched**	157	112	93	69	3
**TOTAL NUMBER OF IDS IN WATER**					
**DotProduct**	734	545	482	387	31
**FracMatched**	734	461	235	89	20
**HyperGeometic**	734	346	134	59	38
**MVH**	734	351	145	60	42
**NumMatches**	734	339	133	54	37
**TicMatched**	734	645	596	502	29

Table S3: Metabolite IDs in Yeast Background

	Score Threshold (%Max Value)				
**MS2 SCORE**	>0%	25%	50%	75%	100%
**YEAST BACKGROUND TRUE POSITIVES**					
**DotProduct**	328	277	247	208	22
**FracMatched**	336	278	180	92	38
**HyperGeometic**	334	231	100	47	23
**MVH**	327	213	93	42	20
**NumMatches**	336	237	102	49	28
**TicMatched**	338	331	319	293	33
**YEAST BACKGROUND FALSE POSITIVES**					
**DotProduct**	366	179	139	95	14
**FracMatched**	358	222	113	40	12
**HyperGeometic**	360	144	40	12	6
**MVH**	367	142	43	15	5
**NumMatches**	358	161	54	19	7
**TicMatched**	356	287	232	178	11
**TOTAL NUMBER OF IDS IN YEAST**					
**DotProduct**	694	456	386	303	36
**FracMatched**	694	500	293	132	50
**HyperGeometic**	694	375	140	59	29
**MVH**	694	355	136	57	25
**NumMatches**	694	398	156	68	35
**TicMatched**	694	618	551	471	44

Figure S5: Metabolomics Isoforms Examples

(A) Ribonolactone [M-H]- RT =13.5 min identification

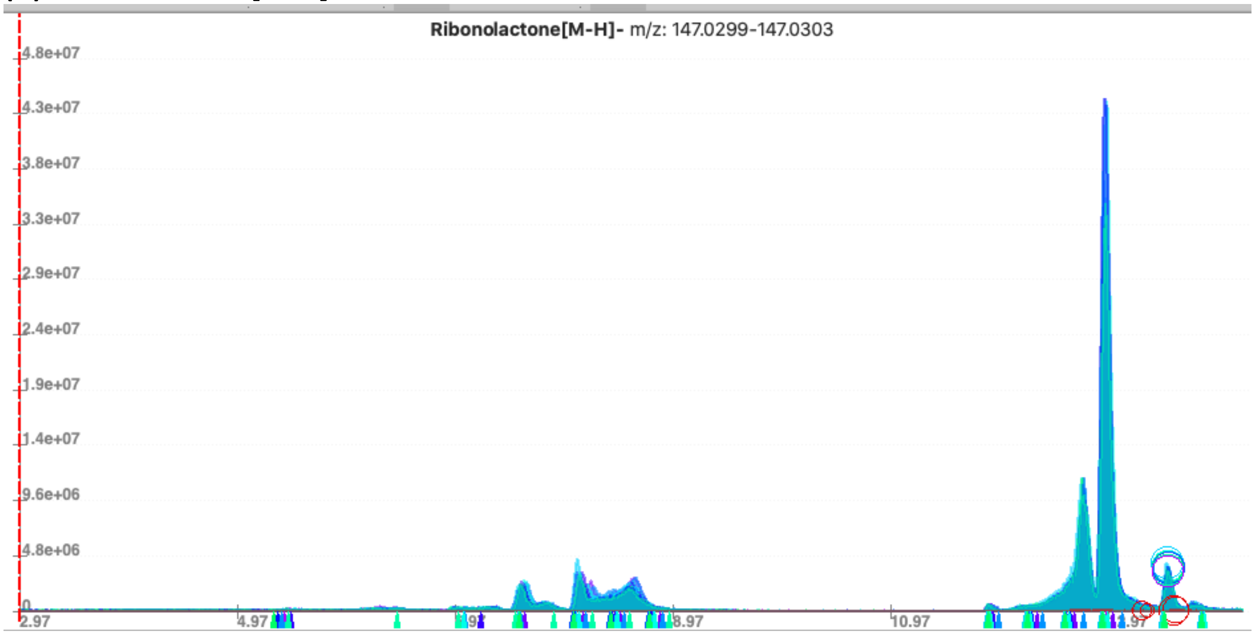

(B) MS/MS spectrum for RT=13.5 min peak group

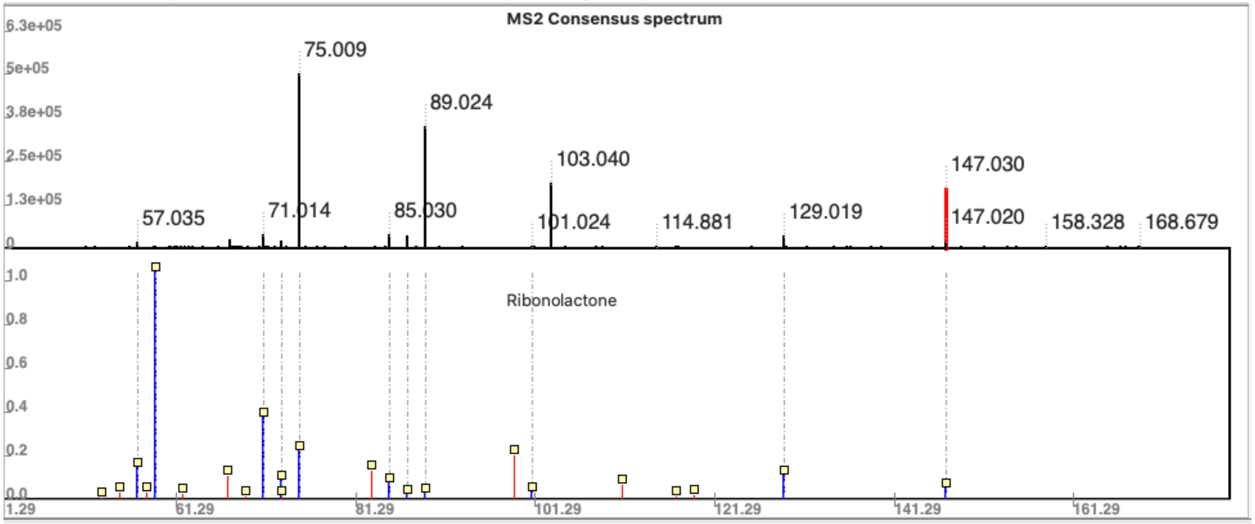

(C) Ribonolactone [M-H]- RT=9 min identification

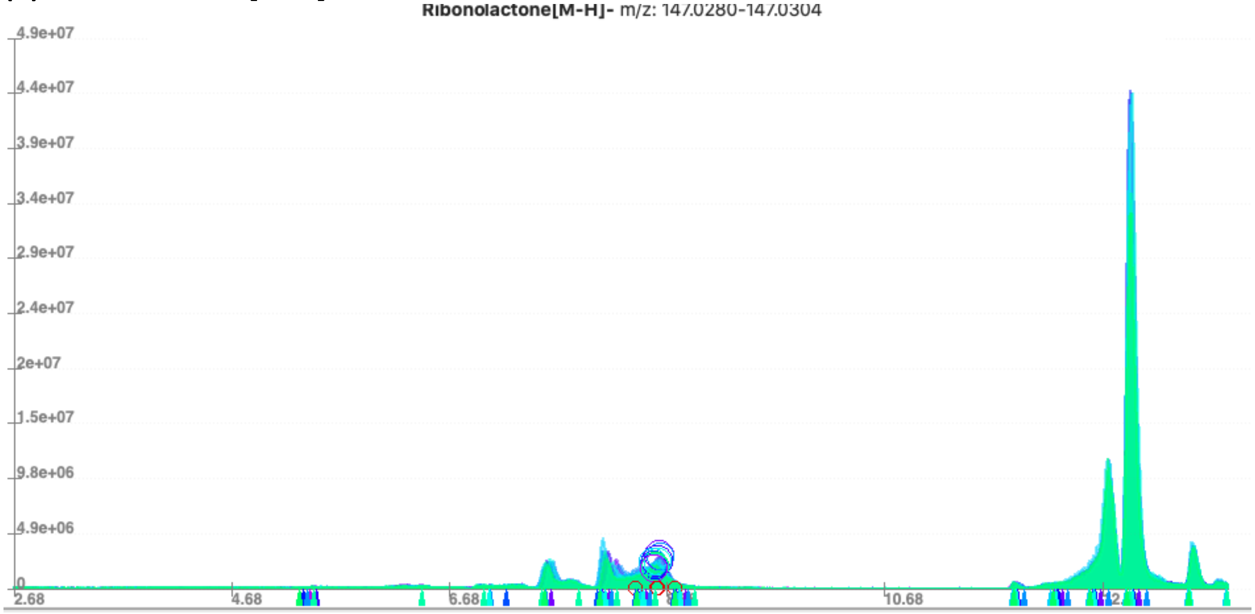

(D) MS/MS spectrum for Rt=9 min peak group

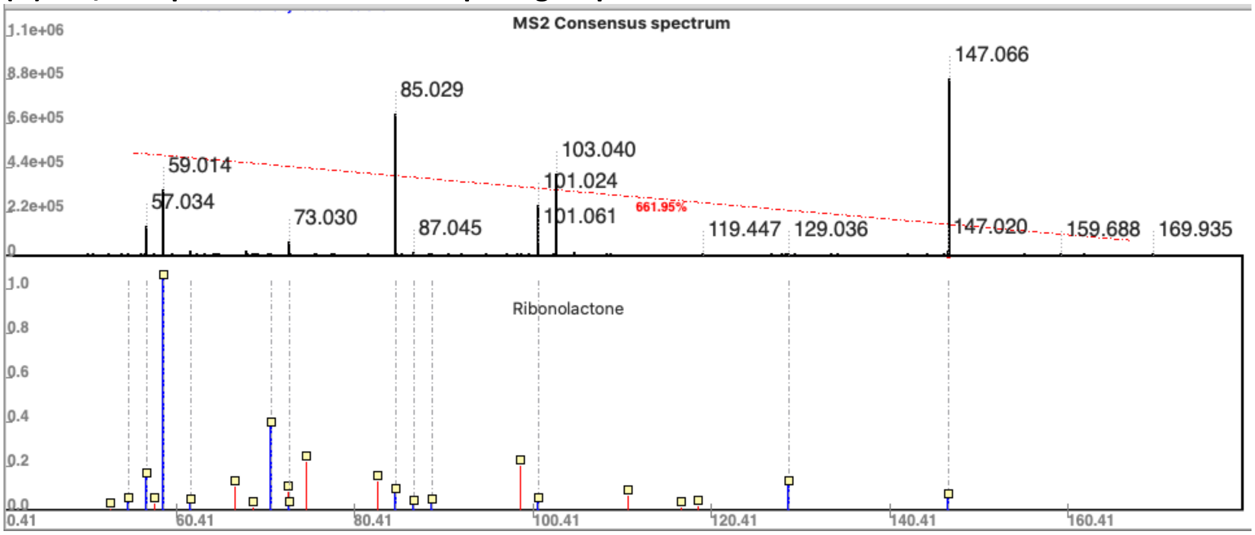

Based on only the matched MS/MS information, it is difficult to tell that one is a correct identification (confirmed with an injected chemical standard), and another is probably a structural isomer with some structural features in common with the true compound of interest. However, the vastly different retention times (9 and 13.5 minutes) indicate that these peak groups must correspond to different compounds.

Dataset S4:

See associated file "Dataset_S4_Metabolomics_Analysis_Script.zip"

Dataset S5:

See associated file "Dataset_S5_Library_Comparisons.zip"
