# Supplementary material for: MAVEN2: An Updated Open-Source Mass Spectrometry Exploration Platform
Source: Metabolites. 2022 Jul 25;12(8):684. doi: 10.3390/metabo12080684 (PMC9330773; doi:10.3390/metabo12080684)
Supplement: Supplementary file 1 [file metabolites-12-00684-s001.zip › metabolites-1827771_proof-supplementary/Dataset_S3_Metabolomics_Example_Analysis.zip!/S6_Metabolomics_Example_Analysis/Metabolomics_Example_Analysis.html]

Metabolomics Example Analysis


# Metabolomics Example Analysis

```
knitr::opts_chunk$set(echo = TRUE, fig.align="center")
```

# Availability on youtube

a video tutorial is available on YouTube, at this URL: Video Tutorial

# Data Processing Steps

## Load Samples

We are loading files that were acquired with data dependent
fragmentation events. There are two groups of files, water sample and
yeast samples spiked in with known standards from SIGMA metabolite
library (see methods). **Click Open, and Select all positive mode
mzXML Files.**

These files may be retrieved from the Metabolights online database,
accession number MTBLS3097

## Recolor Samples and Add Metadata

User can change color, order, grouping of samples in the Samples
Widget, for example, here we are coloring water samples in blue, and
yeast samples in orange. Under “Set”, adjust the set for each sample to
either “yeast” or “water” as appropriate. This can be done by
double-clicking on the “A” next to each sample, and typing in “yeast” or
“water” in its place.

## Save Project Data into mzrollDB File

Now we can save the project information into project file
**example.mzrollDB** . This file is actually an SQLite
database, and will store information about samples, peaks, identified
compounds, and search settings.

## Load Fragmentation Library

MAVEN9 support import of NIST formatted .msp fragmentation libraries.
To import new library select “Import New Library”. This will cache data.
If library already already has been previously imported, simply select
“Load”.

We will assume that the library has not been previously loaded.
Launch the library dialog by clicking the button in the main window
named “Library”. A dialog box will appear. Once the dialog has appeared,
select “Import New Library”, and navigate to the file
“MS-Method-OO2-A\_ZIC-pHILIC\_Polar\_Positive-unnormalized-matched\_single\_energy.msp”,
which is available in the supplementary files. After the file has
imported, it will appear in the list of available libraries.

## View Library Contents

Loading a library populates “Compound Widget” with detailed
information on the compounds contained in a spectral library. This
includes the compound name, formula, adduct form, retention time,
precursor mass, SMILES string, and other metadata.

The Compounds widget may be found my clicking on the “Compounds” tab,
or the “Compound Library” icon on the toolbar on the far right of the
main window.

## Load Adducts

When this library is loaded, all adducts of all compounds featured in
the library are automatically added to the list of available adducts. To
adjust the set of available adducts, you can click on the “Adducts”
button.

In the following dialog that appears, clicking on the “enabled”
header will show all adducts that are currently active in MAVEN. These
adducts will appear in the main window, and will be used for library
searches.

## Examine a Single Compound

For example, lets examine EIC and fragmentation specta for
Glutathione.

Type “Glutathione” in the Compound Name Filter and hit enter. The
filtered list will show 3 compounds, the bottom of which is Glutathione.
Select this Glutathione from the list.

Make sure that the “Fragmentation Widget” is showing. This can be
shown by clicking on the “MS2 Spectra” button on the toolbar on the far
right. Next, in the “Widgets” main program drop-down menu, select “MS2
Scans List”.

Now, zoom in to this large peak by dragging a rectangle over the
peak, starting in the top left corner and moving to the right. Clicking
on the top circle will select all MS2 scans currently associated with
all samples with this peak group, and show a comparison of a consensus
spectrum of this peak group to the library spectrum for Glutathione.

Clicking on individual rows in the MS2 List while holding down CTRL
or SHIFT adjusts the MS2 scans used to build the consensus spectrum.
Clicking on only one row shows that individual scan. The process by
which consensus spectra are generated may be controlled by navigating to
“Options” and choosing the “Spectral Agglomeration” tab.

## Peak Detection

Now we going to search database for compounds that match MS2 spectra.
Clicking on “Peaks” will brings up search parameter dialog. We will use
default settings, and limit our seach to peaks that have MS2 events by
keeping the “Must have MS2” check box checked. We will also click on
“Match Retention Time”, to require that retention time of compound is
within 2 min of annotated retention time of compound.

We will also require that minimum hypertgeometric score match is
>10 with at least 3 fragmentation matches. Click “Find Peaks” to
initiate the search.

Upon completion, search results will appear in the “Detected
Features” widget. Detected features can be sorted by retention time,
precursor mass, etc. For example sorting by “MS2 Score” column lists
best matches first.

Click Save or Press “CTRL + S” to save the results into the mzrollDB
file.

## Peak Group Tagging

To show the power of tagging, we will now tag some the peak groups,
and filter to show only these groups.

Right click on the first peak group and select “Tag Group library”.
click on the second peak group in the list, hold the shift key, and
click the fifth peak group in the list. right-click and select “Tag
group revisit”. Click the sixth group in the list, and hold down CTRL
and type the “g” key. Scroll down to the very end of the table, select
the last row, and click the red “X” button at the top of the “Detected
Features” table. The tooltip for this button will read “Mark Peak as
Bad”. Find the Filter buttonin the “Detected Features” table directly to
the left of the Filter bar. This button will have four smalelr shape
icons in it. Click this button to launch a filter dialog. Once the
dialog appears, uncheck the top row, which will hide all peak groups
that have not been tagged. Click “Apply Filter”.

After carrying out these steps, the peak group table should now look
like this:

Tagged Peak Groups

# Post Processing of Search Results in R

Now that we have analyzed data in MAVEN, we will import this data
into R, where we will perform additional processing.

## Load Data from the Project File (mzrollDB)

There are three main tables in the database.

1. **samples** - information about samples, sample
   attributes, sample groups,
2. **peakgroups** - information about detected
   features, matched compound and adduct infomation
3. **peaks** - individual peak data, one per sample
   with information about retention time, peak intensity, width
   etc..

To execute these scripts, you will need to set your working directory
to the folder containing this script. You will also need to install
various dependencies (shown below)

```
install.packages("tidyverse")
install.packages("reshape2")
install.packages("ggplot2")
install.packages("uwot")
install.packages("dbscan")
```

```
library(tidyverse)
library(reshape2)
library(ggplot2)

con <- DBI::dbConnect(RSQLite::SQLite(), dbname = "./example.mzrollDB")

##sample information
samples <- tbl(con, "samples")
SAMPLES <- samples %>% select(sampleId,samplename=name) %>% collect()
SAMPLES$sampleType <- "sample"
SAMPLES$sampleType[ grepl("blank",ignore.case = T,SAMPLES$samplename) ] = "blank"

SAMPLES$niceNames=gsub("Pos_standards_yeast_background_...",  "", SAMPLES$samplename)
SAMPLES$niceNames=gsub(".mzXML",  "", SAMPLES$niceNames)

##groups
SAMPLES$sampleSet= "";
SAMPLES$sampleSet[ grepl("yeast",ignore.case = T,SAMPLES$samplename) ] = "yeast_spikein"
SAMPLES$sampleSet[ grepl("water",ignore.case = T,SAMPLES$samplename) ] = "water_spikein"
SAMPLES$sampleSet[ grepl("blank",ignore.case = T,SAMPLES$samplename) ] = "blank"

#peakgroup information
peakgroups <- tbl(con, "peakgroups") 
PEAKGROUPS = peakgroups %>% collect()

##peak information
PEAKS=tbl(con, "peaks") %>% left_join(peakgroups, by="groupId") %>%  left_join(samples, by="sampleId") %>% collect()
```

## Group Statistics

```
groupStats=PEAKS %>% left_join(SAMPLES) %>% group_by(groupId,sampleSet) %>%
          summarise(log2intensity=log2(mean(peakAreaTop)),
                    groupMz = median(peakMz),
                    groupRt = median(rt),
                    hyperGeomScore=median(ms2Score),
                    npeaks=n()) %>%
          mutate(goodmatch=hyperGeomScore>50)
```

```
## Joining, by = "sampleId"
## `summarise()` has grouped output by 'groupId'. You can override using the
## `.groups` argument.
```

```
groupStats=groupStats %>% left_join(PEAKGROUPS)
```

```
## Joining, by = "groupId"
```

```
ggplot(groupStats,aes(y=log2intensity,x=groupId,size=log2intensity, color=sampleSet)) + geom_point(aes(alpha=goodmatch),show.legend = T) + 
  theme_bw(base_size = 14) +  scale_color_brewer(palette="Set1")
```

```
## Warning: Using alpha for a discrete variable is not advised.
```

## Correlation between Samples

```
GOODSAMPLES=SAMPLES %>% filter(sampleType == "sample");

XX=PEAKS %>% filter(sampleId %in% GOODSAMPLES$sampleId) %>% left_join(SAMPLES) %>% 
                     reshape2::dcast(.,groupId ~ niceNames,
                                     value.var = "peakAreaTop", 
                                     fun.aggregate = median,
                                     fill = 100)
```

```
## Joining, by = "sampleId"
```

```
for(col in 2:ncol(XX)) { XX[,col] = log2(XX[,col]); }
XXcor=cor(XX[,c(-1)],use = "complete.obs")
pheatmap::pheatmap(XXcor)
```

```
#referenceSample="water_01"
#reference=XX[,referenceSample];
#for(col in 2:ncol(XX)) { XX[,col] = log2(XX[,col]/(reference+10)) }
## plot heatmap
```

## UMAP Clustering of Metabolites

```
library("dbscan")
library("uwot")
```

```
## Loading required package: Matrix
```

```
## 
## Attaching package: 'Matrix'
```

```
## The following objects are masked from 'package:tidyr':
## 
##     expand, pack, unpack
```

```
#project compounds
Z.umap = uwot::umap(XX[,-1],n_neighbors = 2,min_dist = 0.03);
umap_layout = data.frame( groupId=XX[1], umap1=Z.umap[,1], umap2= Z.umap[,2]);

#cluster compounds
dbclust=hdbscan(Z.umap,minPts = 3)
umap_layout$umap_cluster= as.factor(dbclust$cluster)

p0=ggplot(umap_layout, aes(umap1,umap2,color=umap_cluster)) + 
    geom_point(size=2) + theme_bw() + theme(legend.position = "none") +
    ggtitle("UMAP Projection")
print(p0)
```

```
complete_groups=PEAKS %>% left_join(SAMPLES) %>% 
  group_by(groupId,sampleSet) %>%
  summarise(nsamples=n(),
            log2intensity=mean(log2(peakAreaTop)),
            variance=sd(log2(peakAreaTop))) %>% 
  reshape2::dcast(.,groupId ~ sampleSet,
                                     value.var = "log2intensity", 
                                     fun.aggregate = max,
                                    fill=)
```

```
## Joining, by = "sampleId"
## `summarise()` has grouped output by 'groupId'. You can override using the
## `.groups` argument.
```

```
## Warning in .fun(.value[0], ...): no non-missing arguments to max; returning -Inf
```

```
ggplot(complete_groups,aes(y=water_spikein,x=blank)) + geom_point() +
  theme_bw(base_size = 14) +  scale_color_brewer(palette="Set1")
```
