# Supplementary material for: MAVEN2: An Updated Open-Source Mass Spectrometry Exploration Platform
Source: Metabolites. 2022 Jul 25;12(8):684. doi: 10.3390/metabo12080684 (PMC9330773; doi:10.3390/metabo12080684)
Supplement: Supplementary file 1 [file metabolites-12-00684-s001.zip › metabolites-1827771_proof-supplementary/Dataset_S1_Lipidomics_Analysis.zip!/S1_Lipidomics_Analysis/S1_Lipidomics_Analysis_Script.html]

MAVEN Paper Lipid Analysis


# MAVEN Paper Lipid Analysis

#### Phillip Seitzer

This script compares lipid IDs from different libraries searched under identical maven searches.  
 To execute this script, please set your working directory to be whatever directory contains this Rmd file.

# Functions

All functions used in this script are defined in this chunk.

```
lipid_components <- function(compound_name) {

  compound_components <- tibble::tibble("compoundName" = compound_name) %>%

    #general compound information
    dplyr::mutate(lipidClass = stringr::str_extract(compoundName,"^.*(?=\\()")) %>%
    dplyr::mutate(compound_name_adduct = stringr::str_extract(compoundName,"(?<=\\) ).*$")) %>%
    dplyr::mutate(plasmalogen_type = stringr::str_extract(compoundName,"[op]-(?=[0-9]+:)")) %>%
    dplyr::mutate(lipidClass_o_p = ifelse(is.na(plasmalogen_type), lipidClass, paste0(plasmalogen_type, lipidClass))) %>%
    dplyr::mutate(lipidClass_plas = ifelse(is.na(plasmalogen_type), lipidClass, paste0("plas-",lipidClass))) %>%

    #Parse all chains.  Also retrieves number of hydroxyl groups on each FA (m=1, d=2, t=3), if available
    dplyr::mutate(sn_chains = stringr::str_extract_all(compoundName,"(?<=[\\(/_])[A-Za-z]?-?[0-9]+:[0-9]+;?O?[0-9]?(?=[\\)/_])")) %>%
    dplyr::mutate(num_sn_chains = sapply(sn_chains, function(x){length(x)})) %>%
    dplyr::mutate(num_unique_sn_chains = sapply(sn_chains, function(x){x %>% unique() %>% length()})) %>%

    #sn1
    dplyr::mutate(sn1 = sapply(sn_chains, function(x){x[1]})) %>%
    dplyr::mutate(FA1 = stringr::str_extract(sn1,"[0-9]+:[0-9]+")) %>%
    dplyr::mutate(single_1 = as.numeric(stringr::str_extract(FA1, ".*(?=:)"))) %>%
    dplyr::mutate(single_1 = ifelse(is.na(single_1), 0, single_1)) %>%
    dplyr::mutate(double_1 = as.numeric(stringr::str_extract(FA1,"(?<=:).*"))) %>%
    dplyr::mutate(double_1 = ifelse(is.na(double_1), 0, double_1)) %>%
    dplyr::mutate(double_1 = ifelse((!is.na(plasmalogen_type) & plasmalogen_type == "p-"), double_1+1, double_1)) %>%
    dplyr::mutate(OH_1 = case_when(
                           grepl("^m", sn1) ~ 1,
                           grepl("^d", sn1) ~ 2,
                           grepl("^t", sn1) ~ 3,
                           grepl(";O4", sn1) ~ 4,
                           grepl(";O3", sn1) ~ 3,
                           grepl(";O2", sn1) ~ 2,
                           grepl(";O1", sn1) ~ 1,
                           grepl(";O", sn1) ~ 1,
                           TRUE ~ 0)) %>%

    # sn2
    dplyr::mutate(sn2=sapply(sn_chains, function(x){x[2]})) %>%
    dplyr::mutate(FA2 = stringr::str_extract(sn2,"[0-9]+:[0-9]+")) %>%
    dplyr::mutate(single_2 = as.numeric(stringr::str_extract(FA2, ".*(?=:)"))) %>%
    dplyr::mutate(single_2 = ifelse(is.na(single_2), 0, single_2)) %>%
    dplyr::mutate(double_2 = as.numeric(stringr::str_extract(FA2,"(?<=:).*"))) %>%
    dplyr::mutate(double_2 = ifelse(is.na(double_2), 0, double_2)) %>%
    dplyr::mutate(OH_2 = case_when(
                          grepl("^m", sn2) ~ 1,
                          grepl("^d", sn2) ~ 2,
                          grepl("^t", sn2) ~ 3,
                          grepl(";O4", sn2) ~ 4,
                          grepl(";O3", sn2) ~ 3,
                          grepl(";O2", sn2) ~ 2,
                          grepl(";O1", sn2) ~ 1,
                          grepl(";O", sn2) ~ 1,
                          TRUE ~ 0)) %>%

    # sn3 (for TGs)
    dplyr::mutate(sn3 = sapply(sn_chains, function(x){x[3]})) %>%
    dplyr::mutate(FA3 = stringr::str_extract(sn3,"[0-9]+:[0-9]+")) %>%
    dplyr::mutate(single_3 = as.numeric(stringr::str_extract(FA3, ".*(?=:)"))) %>%
    dplyr::mutate(single_3 = ifelse(is.na(single_3), 0, single_3)) %>%
    dplyr::mutate(double_3 = as.numeric(stringr::str_extract(FA3,"(?<=:).*"))) %>%
    dplyr::mutate(double_3 = ifelse(is.na(double_3), 0, double_3)) %>%
    dplyr::mutate(OH_3 = case_when(
                          grepl("^m", sn3) ~ 1,
                          grepl("^d", sn3) ~ 2,
                          grepl("^t", sn3) ~ 3,
                          grepl(";O4", sn3) ~ 4,
                          grepl(";O3", sn3) ~ 3,
                          grepl(";O2", sn3) ~ 2,
                          grepl(";O1", sn3) ~ 1,
                          grepl(";O", sn3) ~ 1,
                          TRUE ~ 0)) %>%
    
    #sn4 (for Cardiolipins CLs)
    dplyr::mutate(sn4 = sapply(sn_chains, function(x){x[4]})) %>%
    dplyr::mutate(FA4 = stringr::str_extract(sn4,"[0-9]+:[0-9]+")) %>%
    dplyr::mutate(single_4 = as.numeric(stringr::str_extract(FA4, ".*(?=:)"))) %>%
    dplyr::mutate(single_4 = ifelse(is.na(single_4), 0, single_4)) %>%
    dplyr::mutate(double_4 = as.numeric(stringr::str_extract(FA4,"(?<=:).*"))) %>%
    dplyr::mutate(double_4 = ifelse(is.na(double_4), 0, double_4)) %>%
    dplyr::mutate(OH_4 = case_when(
                          grepl("^m", sn4) ~ 1,
                          grepl("^d", sn4) ~ 2,
                          grepl("^t", sn4) ~ 3,
                          grepl(";O4", sn4) ~ 4,
                          grepl(";O3", sn4) ~ 3,
                          grepl(";O2", sn4) ~ 2,
                          grepl(";O1", sn4) ~ 1,
                          grepl(";O", sn4) ~ 1,
                          TRUE ~ 0)) %>%

    # finally, put together all info from chains to describe summed composition
    # sometimes, the compound is already reported as a summed composition. In that case, return the original name.
    dplyr::mutate(total_single = ifelse(num_sn_chains==0, as.numeric(stringr::str_extract(compoundName,"(?<=\\()[A-Za-z]?-?[0-9]+(?=:[0-9]+,)")) ,(single_1+single_2+single_3+single_4))) %>%
    dplyr::mutate(total_single = ifelse(is.na(total_single), 0, total_single)) %>%
    dplyr::mutate(total_double = ifelse(num_sn_chains==0, as.numeric(stringr::str_extract(compoundName,"(?<=:)[0-9]+(?=,)")), (double_1+double_2+double_3+double_4))) %>%
    dplyr::mutate(total_double = ifelse(is.na(total_double), 0, total_double)) %>%
    dplyr::mutate(total_OH = ifelse(num_sn_chains==0, as.numeric(stringr::str_extract(compoundName,"(?<=,)[0-9]+(?=-)")), OH_1+OH_2+OH_3+OH_4)) %>%
    dplyr::mutate(total_OH = ifelse(is.na(total_OH), 0, total_OH)) %>%
    dplyr::mutate(sumComposition = case_when(
      num_sn_chains > 0 & total_OH == 0 ~ glue::glue('{class}({plas}{single}:{double})',
                                               class = lipidClass,
                                               plas = ifelse(is.na(plasmalogen_type),"",plasmalogen_type),
                                               single = total_single,
                                               double = ifelse((!is.na(plasmalogen_type) & plasmalogen_type=="p-"), (total_double-1), total_double)),
      num_sn_chains >0 & total_OH == 1 ~ glue::glue('{class}({plas}{single}:{double};O)',
                                                   class = lipidClass,
                                                   plas = ifelse(is.na(plasmalogen_type),"",plasmalogen_type),
                                                   single = total_single,
                                                   double = ifelse((!is.na(plasmalogen_type) & plasmalogen_type=="p-"), (total_double-1), total_double)),
      num_sn_chains > 0 & total_OH > 1 ~ glue::glue('{class}({plas}{single}:{double};O{num_OH})',
                                               class = lipidClass,
                                               plas = ifelse(is.na(plasmalogen_type),"",plasmalogen_type),
                                               single = total_single,
                                               double = ifelse((!is.na(plasmalogen_type) & plasmalogen_type=="p-"), (total_double-1), total_double),
                                               num_OH = total_OH),
      num_sn_chains == 0 ~ compoundName)) %>%
    dplyr::mutate(etherPlasmalogenCompoundName = ifelse(is.na(plasmalogen_type), compoundName,
                                            glue::glue('{class}({single}:{double}e/{sn2_chain})',
                                                       class = lipidClass,
                                                       single = single_1,
                                                       double = double_1,
                                                       sn2_chain = FA2))) %>%
    dplyr::mutate(etherPlasmalogenSumComposition = ifelse(is.na(plasmalogen_type), sumComposition,
                                             glue::glue('{class}({single}:{double}e)',
                                                        class = lipidClass,
                                                        single=total_single,
                                                        double=total_double)))

  compound_components
}

reformat_calico_results <- function(calico_tbl){
  calico_tbl_reformatted <- calico_tbl %>%
  dplyr::select(medMz, medRt, compound, adductName, compoundLipidClass, hyperGeomScore, fragNumIonsMatched) %>%
  dplyr::mutate(compound = as.character(compound)) %>%
  dplyr::rename(calico_compound = compound) %>%
  dplyr::rename(calico_adduct = adductName) %>%
  dplyr::rename(calico_hyperGeomScore = hyperGeomScore) %>%
  dplyr::rename(calico_fragNumIonsMatched = fragNumIonsMatched) %>%
  dplyr::rename(calico_lipidClass = compoundLipidClass) %>%
  dplyr::mutate(calico_lipidClass = ifelse(calico_lipidClass == "", "unidentified", as.character(calico_lipidClass))) %>%
  dplyr::mutate(calico_lipidClass = ifelse(calico_lipidClass == "PE" & grepl("[op]-", calico_compound), "Alkyl_PE", calico_lipidClass)) %>%
  dplyr::mutate(calico_lipidClass = ifelse(calico_lipidClass == "PC" & grepl("[op]-", calico_compound), "Alkyl_PC", calico_lipidClass))
  
  calico_lipid_components <- lipid_components(calico_tbl_reformatted$calico_compound %>% unique()) %>%
    dplyr::select(compoundName, etherPlasmalogenCompoundName, etherPlasmalogenSumComposition)
  
  calico_tbl_reformatted_w_lipid_data <- dplyr::inner_join(calico_tbl_reformatted, calico_lipid_components, by = c("calico_compound"="compoundName")) %>%
    dplyr::rename(calico_sumComposition = etherPlasmalogenSumComposition) %>%
    dplyr::select(-calico_compound) %>%
    dplyr::rename(calico_compound = etherPlasmalogenCompoundName) %>%
    
    # Avoid 0-length chain acyl chains for agreement with MS-DIAL libraries
    dplyr::mutate(calico_compound_adj = ifelse(calico_lipidClass %in% c("LPC","LPE"), calico_sumComposition, calico_compound)) %>%
    
    dplyr::mutate(calico_compound_adj = ifelse(calico_compound_adj == "", "unidentified", calico_compound_adj)) %>%
    
    dplyr::select(medMz, medRt,
                  calico_compound,
                  calico_lipidClass, calico_sumComposition, calico_compound_adj, calico_adduct, calico_hyperGeomScore, calico_fragNumIonsMatched
                  )
}

msdial_compound_info <- function(msdial_tbl) {
  
  msdial_tbl_reformatted <- msdial_tbl %>%
    dplyr::mutate(compound = gsub("\r", "", compound)) %>%
    dplyr::mutate(msdial_adduct = gsub("\r", "", msdial_adduct)) %>%
    dplyr::mutate(name_components = stringr::str_split(compound, ";")) %>%
    dplyr::mutate(msdial_compound = str_squish(sapply(name_components, function(x){as.character(x[2])}))) %>%
    dplyr::mutate(msdial_compound = ifelse(is.na(msdial_compound), "", msdial_compound)) %>%
    dplyr::mutate(msdial_compound_alt = str_squish(sapply(name_components, function(x){as.character(x[1])}))) %>%
    dplyr::mutate(msdial_compound = ifelse(msdial_compound == msdial_adduct, msdial_compound_alt, msdial_compound)) %>%
    dplyr::rename(original_compound = compound) %>%
    dplyr::select(-name_components, -msdial_compound_alt) %>%
    dplyr::rename(msdial_lipidClass = lipidClass) %>%
    dplyr::mutate(msdial_lipidClass = gsub("\r","", msdial_lipidClass)) %>%
    dplyr::mutate(compound_components = stringr::str_split(msdial_compound, " ")) %>%
    dplyr::mutate(compound_acyl_chains = sapply(compound_components, function(x){as.character(x[2])})) %>%
    dplyr::mutate(compound_acyl_chains = gsub("\\-","/", compound_acyl_chains)) %>%
    dplyr::mutate(compound_acyl_chains = gsub("\\+",";", compound_acyl_chains)) %>%
    dplyr::mutate(compound_acyl_chains = gsub("\\([0-9]Cyc\\)","", compound_acyl_chains)) %>%
    dplyr::mutate(compound_acyl_chains = gsub("e","", compound_acyl_chains)) %>%
    dplyr::mutate(msdial_lipidClass_adj = case_when(
      is.na(msdial_lipidClass) ~ "unidentified",
      msdial_lipidClass == "TAG" ~ "TG",
      msdial_lipidClass == "EtherPC" ~ "Alkyl_PC",
      msdial_lipidClass == "Cer_NS" ~ "Ceramide",
      msdial_lipidClass == "Cer_NDS" ~ "Ceramide",
      msdial_lipidClass == "Cer_NP" ~ "Ceramide",
      msdial_lipidClass == "Cer_AS" ~ "Ceramide",
      msdial_lipidClass == "GlcCer_NS" ~ "HexCer",
      msdial_lipidClass == "GlcCer_AP" ~ "HexCer",
      msdial_lipidClass == "GlcCer_NDS" ~ "HexCer",
      msdial_lipidClass == "EtherPE" ~ "Alkyl_PE",
      msdial_lipidClass == "EtherPC" ~ "Alkyl_PC",
      msdial_lipidClass == "GM3" ~ "AcGM3",
      msdial_lipidClass == "DAG" ~ "DG",
      TRUE==TRUE ~ msdial_lipidClass
    )) %>%
    dplyr::mutate(msdial_compound_adj = case_when(
      msdial_lipidClass_adj == "unidentified" ~ "unidentified",
      msdial_lipidClass_adj == "Alkyl_PC" ~ paste0("PC(o-", compound_acyl_chains,")"),
      msdial_lipidClass_adj == "Alkyl_PE" ~ paste0("PE(o-", compound_acyl_chains,")"),
      TRUE==TRUE ~ paste0(msdial_lipidClass_adj,"(",compound_acyl_chains,")")
    )) %>%
    dplyr::select(-compound_components)
}

reformat_msdial_results <- function(msdial_tbl) {
  msdial_tbl_reformatted <- msdial_tbl %>%
  dplyr::select(medMz, medRt, compound, adductName, hyperGeomScore, fragNumIonsMatched, category) %>%
  dplyr::mutate(msdial_adduct = as.character(adductName)) %>%
  dplyr::mutate(name_components = stringr::str_split(compound, ";")) %>%
  dplyr::mutate(msdial_compound = str_squish(sapply(name_components, function(x){as.character(x[2])}))) %>%
  dplyr::mutate(msdial_compound = ifelse(is.na(msdial_compound), "", msdial_compound)) %>%
  dplyr::mutate(msdial_compound_alt = str_squish(sapply(name_components, function(x){as.character(x[1])}))) %>%
  dplyr::mutate(msdial_compound = ifelse(msdial_compound == msdial_adduct, msdial_compound_alt, msdial_compound)) %>%
  dplyr::select(-name_components, -compound, -adductName, -msdial_compound_alt) %>%
  dplyr::rename(msdial_hyperGeomScore = hyperGeomScore) %>%
  dplyr::rename(msdial_fragNumIonsMatched = fragNumIonsMatched) %>%
  dplyr::mutate(category = ifelse(category=="","unidentified", as.character(category))) %>%
  dplyr::rename(msdial_lipidClass = category) %>%
  dplyr::mutate(compound_components = stringr::str_split(msdial_compound, " ")) %>%
  dplyr::mutate(compound_acyl_chains = sapply(compound_components, function(x){as.character(x[2])})) %>%
  dplyr::mutate(compound_acyl_chains = gsub("\\-","/", compound_acyl_chains)) %>%
  dplyr::mutate(compound_acyl_chains = gsub("\\+",";", compound_acyl_chains)) %>%
  dplyr::mutate(compound_acyl_chains = gsub("\\([0-9]Cyc\\)","", compound_acyl_chains)) %>%
  dplyr::mutate(compound_acyl_chains = gsub("e","", compound_acyl_chains)) %>%
  dplyr::mutate(msdial_lipidClass_adj = case_when(
    is.na(msdial_lipidClass) ~ "unidentified",
    msdial_lipidClass == "TAG" ~ "TG",
    msdial_lipidClass == "EtherPC" ~ "Alkyl_PC",
    msdial_lipidClass == "Cer_NS" ~ "Ceramide",
    msdial_lipidClass == "Cer_NDS" ~ "Ceramide",
    msdial_lipidClass == "Cer_NP" ~ "Ceramide",
    msdial_lipidClass == "Cer_AS" ~ "Ceramide",
    msdial_lipidClass == "GlcCer_NS" ~ "HexCer",
    msdial_lipidClass == "GlcCer_AP" ~ "HexCer",
    msdial_lipidClass == "GlcCer_NDS" ~ "HexCer",
    msdial_lipidClass == "EtherPE" ~ "Alkyl_PE",
    msdial_lipidClass == "EtherPC" ~ "Alkyl_PC",
    msdial_lipidClass == "GM3" ~ "AcGM3",
    msdial_lipidClass == "DAG" ~ "DG",
    TRUE==TRUE ~ msdial_lipidClass
  )) %>%
  dplyr::mutate(msdial_compound_adj = case_when(
        msdial_lipidClass_adj == "unidentified" ~ "unidentified",
        msdial_lipidClass_adj == "Alkyl_PC" ~ paste0("PC(o-", compound_acyl_chains,")"),
        msdial_lipidClass_adj == "Alkyl_PE" ~ paste0("PE(o-", compound_acyl_chains,")"),
        TRUE==TRUE ~ paste0(msdial_lipidClass_adj,"(",compound_acyl_chains,")")
    ))
  
  # TODO: currently calling calicomics, which is not released. Release calicomics or factor out
  msdial_lipid_components <- lipid_components(msdial_tbl_reformatted$msdial_compound_adj %>% unique()) %>%
    dplyr::select(compoundName, etherPlasmalogenCompoundName, etherPlasmalogenSumComposition)
  
  msdial_tbl_reformatted_w_lipid_data <- dplyr::inner_join(msdial_tbl_reformatted, msdial_lipid_components, by = c("msdial_compound_adj"="compoundName")) %>%
    dplyr::rename(msdial_sumComposition_adj = etherPlasmalogenSumComposition) %>%
    dplyr::select(-msdial_compound_adj) %>%
    dplyr::rename(msdial_compound_adj = etherPlasmalogenCompoundName) %>%
    dplyr::select(medMz, medRt,
                  msdial_compound,
                  msdial_lipidClass_adj, msdial_sumComposition_adj, msdial_compound_adj, msdial_adduct, msdial_hyperGeomScore, msdial_fragNumIonsMatched)
  
  msdial_tbl_reformatted_w_lipid_data
}

combine_search_results <- function(calico_tbl, msdial_tbl) {
  calico_msdial_combined <- dplyr::inner_join(calico_tbl, msdial_tbl, by = c("medMz","medRt")) %>%
  dplyr::select(medMz, medRt,
                calico_lipidClass, calico_sumComposition, calico_compound_adj, calico_adduct, calico_hyperGeomScore, calico_fragNumIonsMatched,
                msdial_lipidClass_adj, msdial_sumComposition_adj, msdial_compound_adj, msdial_adduct, msdial_hyperGeomScore, msdial_fragNumIonsMatched,
                calico_compound, msdial_compound # retain original compound strings for debugging
                ) %>%
  dplyr::mutate(annotation_type = case_when(
    
    calico_compound_adj == "unidentified" & msdial_compound_adj == "unidentified" ~ "neither",
    calico_compound_adj != "unidentified" & msdial_compound_adj == "unidentified" ~ "calico only",
    calico_compound_adj == "unidentified" & msdial_compound_adj != "unidentified" ~ "msdial only",

    # Indicates that an annotation was rendered for both features
    calico_compound_adj != "unidentified" & msdial_compound_adj != "unidentified" ~ "both")) %>%
    
    dplyr::mutate(is_agreement_class = annotation_type == "both" & calico_lipidClass == msdial_lipidClass_adj) %>%
    dplyr::mutate(is_agreement_adduct = annotation_type == "both" & calico_adduct == msdial_adduct) %>%
    dplyr::mutate(is_agreement_sumComposition = annotation_type == "both" & calico_sumComposition == msdial_sumComposition_adj) %>%
    dplyr::mutate(is_agreement_compound = annotation_type == "both" & calico_compound_adj == msdial_compound_adj) %>%
    dplyr::arrange(medMz, medRt)
}

combine_modes <- function(calico_msdial_pos_tbl, calico_msdial_neg_tbl) {
  calico_msdial_pos_mode <- calico_msdial_pos_tbl %>% dplyr::mutate(mode = "positive ionization mode")
  calico_msdial_neg_mode <- calico_msdial_neg_tbl %>% dplyr::mutate(mode = "negative ionization mode")
  calico_msdial_combined_modes <- rbind(calico_msdial_pos_mode, calico_msdial_neg_mode)
}

search_results <- function(calico_neg_mode_results_raw,
                           calico_pos_mode_results_raw,
                           msdial_neg_mode_results_raw,
                           msdial_pos_mode_results_raw) {
  
  calico_neg_results <- reformat_calico_results(calico_neg_mode_results_raw)
  calico_pos_results <- reformat_calico_results(calico_pos_mode_results_raw)
  msdial_neg_results <- reformat_msdial_results(msdial_neg_mode_results_raw)
  msdial_pos_results <- reformat_msdial_results(msdial_pos_mode_results_raw)
  
  calico_msdial_pos_combined <- combine_search_results(calico_pos_results, msdial_pos_results)
  calico_msdial_neg_combined <- combine_search_results(calico_neg_results, msdial_neg_results)
  
  results <- combine_modes(calico_msdial_pos_combined, calico_msdial_neg_combined)
}

plot_dist <- function(both_scores_subset, plot_title="", b_width=1, is_density=FALSE) {
  
  both_scores_subset_part <- both_scores_subset %>% dplyr::select(medMz, medRt, calico_hyperGeomScore, msdial_hyperGeomScore) %>%
    dplyr::rename(calico = calico_hyperGeomScore, msdial = msdial_hyperGeomScore) %>%
    tidyr::pivot_longer(., cols = c(calico, msdial), names_to = "library", values_to = "score")
  
  if (is_density) {
    p1 <- ggplot(both_scores_subset_part, aes(score, fill= library)) +
      geom_density(alpha = 0.5) +
      ggtitle(plot_title)
    
  } else {
    p1 <- ggplot(both_scores_subset_part, aes(score, fill= library)) +
      geom_histogram(binwidth = b_width, alpha = 0.5, position="dodge") +
      ggtitle(plot_title)
  }
  p1
}

count_AD <- function(AD_results, score_delta=1, min_score=0, max_score=100) {
  
  N <- (max_score-min_score+1) / score_delta
  count_results <- tibble::tibble(score=numeric(N),
                                  num_agree=numeric(N),
                                  agreement_fraction=numeric(N),
                                  num_disagree=numeric(N),
                                  disagreement_fraction=numeric(N),
                                  fraction_diff=numeric(N))
  
  total_agreement <- AD_results %>% dplyr::filter(agreement_type == "agreement") %>% nrow()
  total_disagreement <- AD_results %>% dplyr::filter(agreement_type == "disagreement") %>% nrow()
  
  threshold <- min_score
  
  row_num <- 1
  while(threshold <= max_score) {
    
  AD_results_sum <- AD_results %>% dplyr::filter(score >= threshold) %>%
    dplyr::group_by(agreement_type) %>%
    dplyr::mutate(count = n()) %>%
    dplyr::select(agreement_type, count) %>%
    unique()
  
  n_agree <- 0
  n_disagree <- 0
  
  AD_results_sum_agree <- AD_results_sum %>% dplyr::filter(agreement_type == "agreement")
  AD_results_sum_disagree <- AD_results_sum %>% dplyr::filter(agreement_type == "disagreement")
  
  if (nrow(AD_results_sum_agree) > 0) {
    n_agree <- AD_results_sum_agree$count[1]
  }
  
  if (nrow(AD_results_sum_disagree) > 0) {
    n_disagree <- AD_results_sum_disagree$count[1]
  }
  
  agreement_fraction <- n_agree / total_agreement
  disagreement_fraction <- n_disagree / total_disagreement
  fraction_diff = agreement_fraction-disagreement_fraction
  
  AD_results_sum$count[1]
  count_results[row_num,] <- list(
    "score"=threshold,
    "num_agree"=n_agree,
    "agreement_fraction"=agreement_fraction,
    "num_disagree"=n_disagree,
    "disagreement_fraction"=disagreement_fraction,
    "fraction_diff"=fraction_diff)
  
  threshold <- threshold + score_delta
  row_num <- row_num + 1
  
  }
  
  count_results
}
```

# Import

## MAVEN Results

MAVEN was launched. A sample set and library was loaded.  
 Two sample sets were available, the "internal set" and "external set" described  
 in depth in the corresponding manuscript.  
 The "internal set" is also referred to as the "thermo" set, because it was generated  
 on a Thermo Q Exactive Plus mass spectrometer.  
 The "external set" is also referred to as the "agilent" set, becuase it was generated  
 on an Agilent 6546 QTOF mass spectrometer.  
 Each sample set was generated in both positive and negative ionization mode.  
 For each of these 4 sample collections, the data was searched in Maven against either  
 the Calico or MS-DIAL spectral library of corresponding ionization mode.  
 The following MAVEN search parameters were used:  
 At least 65% of samples had to have a "good" peak group,  
 where "good" was a quality score of >= 0.5 according to the default classifier model.  
 peak groups that did not match to compounds were retained.  
 compounds did not have to have to match any fragments, nor have any score threshold.  
 All other search parameters were set to their default values.  
 Once search results appeared, they were exported into csv files, using the "export"  
 feature available in MAVEN.

```
results_dir_series2 <- "search_results/"

thermo_calico_pos <- read.csv(paste(results_dir_series2, "20210210-thermo-calico-pos.csv", sep="/"))
thermo_msdial_pos <- read.csv(paste(results_dir_series2, "20210210-thermo-msdial-pos.csv", sep="/"))
thermo_calico_neg <- read.csv(paste(results_dir_series2, "20210210-thermo-calico-neg.csv", sep="/"))
thermo_msdial_neg <- read.csv(paste(results_dir_series2, "20210210-thermo-msdial-neg.csv", sep="/"))

agilent_calico_pos <- read.csv(paste(results_dir_series2, "20210210-agilent-calico-pos.csv", sep="/"))
agilent_msdial_pos <- read.csv(paste(results_dir_series2, "20210210-agilent-msdial-pos.csv", sep="/"))
agilent_calico_neg <- read.csv(paste(results_dir_series2, "20210210-agilent-calico-neg.csv", sep="/"))

agilent_msdial_neg <- read.csv(paste(results_dir_series2, "20210218-agilent-msdial-neg-v2.csv", sep="/"))
```

## Lipid libraries

Load library data for Calico and MS-DIAL lipid libraries.  
 These libraries were imported as msp files and exported into an .rds structure for faster reloading.  
 To recover the msp files, please see the instructions associated with the manuscript.  
 The MS-DIAL libraries were retrieved from the library as-is, with the exception that the acetate adduct was   
 altered from [M+Hac-H]- to [M+AcOH-H]-.

```
lipid_libraries <- readRDS("lipid_libraries.rds")

msdial_neg <- lipid_libraries$msdial_neg
msdial_pos <- lipid_libraries$msdial_pos
calico_neg <- lipid_libraries$calico_neg
calico_pos <- lipid_libraries$calico_pos
```

# Agreements and Disagreements Analysis

## Create Single unified dataset

```
thermo_results <- search_results(
  calico_neg_mode_results_raw = thermo_calico_neg,
  calico_pos_mode_results_raw = thermo_calico_pos,
  msdial_neg_mode_results_raw = thermo_msdial_neg,
  msdial_pos_mode_results_raw = thermo_msdial_pos) %>%
  dplyr::mutate(dataset="thermo")

agilent_results <- search_results(
  calico_neg_mode_results_raw = agilent_calico_neg,
  calico_pos_mode_results_raw = agilent_calico_pos,
  msdial_neg_mode_results_raw = agilent_msdial_neg,
  msdial_pos_mode_results_raw = agilent_msdial_pos) %>%
  dplyr::mutate(dataset="agilent")

all_scores_results <- rbind(thermo_results, agilent_results)
```

## Enumerate Types of Agreement

```
# Annotation Types: both
all_scores_both_annotated <- all_scores_results %>% dplyr::filter(annotation_type == "both")

# Agreements
# Perfect agreement
all_scores_results_compound_agreement <- all_scores_both_annotated %>%
  dplyr::filter(is_agreement_class & is_agreement_adduct & is_agreement_sumComposition & is_agreement_compound)

# sum composition or better agreement
all_scores_results_composition_agreement <- all_scores_both_annotated %>%
  dplyr::filter(is_agreement_class & is_agreement_adduct & is_agreement_sumComposition)

# class + adduct agreement or better
all_scores_results_class_and_adduct_agreement <- all_scores_both_annotated %>%
  dplyr::filter(is_agreement_class & is_agreement_adduct)

# class agreement or better
all_scores_results_class_agreement <- all_scores_both_annotated %>%
  dplyr::filter(is_agreement_class)

# Disagreements
all_scores_disagreement <- all_scores_both_annotated %>%
  dplyr::filter(!is_agreement_class & !is_agreement_adduct & !is_agreement_sumComposition & !is_agreement_compound)
```

## Annotations Agreements and Disagreements

```
agreements <- all_scores_results_compound_agreement %>% dplyr::mutate(agreement_type = "agreement")
disagreements <- all_scores_disagreement %>% dplyr::mutate(agreement_type = "disagreement")

agreements_and_disagreements <- rbind(agreements, disagreements) %>%
  dplyr::select(calico_hyperGeomScore, msdial_hyperGeomScore, agreement_type) %>%
  dplyr::rename(calico = calico_hyperGeomScore, msdial = msdial_hyperGeomScore) %>%
  tidyr::pivot_longer(., cols = c(calico, msdial), names_to = "library", values_to = "score") %>%
  dplyr::mutate(type = paste(library, agreement_type, sep=" "))

AD_together <- agreements_and_disagreements %>%
  dplyr::mutate(Type = ifelse(grepl("disagreement", type), "disagreement", "agreement")) %>%
  dplyr::mutate(Library = ifelse(library=="msdial", "MS-DIAL Spectral Library","Calico Spectral Library"))

calico_AD <- agreements_and_disagreements %>% dplyr::filter(library == "calico")
msdial_AD <- agreements_and_disagreements %>% dplyr::filter(library == "msdial")

calico_AD_counts <- count_AD(calico_AD, score_delta=0.1)
msdial_AD_counts <- count_AD(msdial_AD, score_delta=0.1)

calico_AD_counts_w_type <- calico_AD_counts %>% dplyr::rename(calico_fraction_diff = fraction_diff)
msdial_AD_counts_w_type <- msdial_AD_counts %>% dplyr::rename(msdial_fraction_diff = fraction_diff)

both_series <- dplyr::inner_join(calico_AD_counts_w_type, msdial_AD_counts_w_type, by = c("score"))
```

## Agreement and Disagreement Optimal Thresholds

```
geom_thresh_data <- ggplot_build(ggplot(both_series, aes(x=score)) +
  geom_smooth(aes(y=calico_fraction_diff), color="blue") +
  geom_smooth(aes(y=msdial_fraction_diff), color="red")) 
#> `geom_smooth()` using method = 'gam' and formula 'y ~ s(x, bs = "cs")'
#> `geom_smooth()` using method = 'gam' and formula 'y ~ s(x, bs = "cs")'

calico_diff_fit <- geom_thresh_data$data[[1]]
calico_max_pos <- which(calico_diff_fit$y == max(calico_diff_fit$y))
optimal_calico_score_threshold <- round(calico_diff_fit$x[calico_max_pos], digits=1)

msdial_diff_fit <- geom_thresh_data$data[[2]]
msdial_max_pos <- which(msdial_diff_fit$y==max(msdial_diff_fit$y))
optimal_msdial_score_threshold <- round(msdial_diff_fit$x[msdial_max_pos], digits=1)
```

## Threshold results

```
all_scores_results_thresholded <- all_scores_results %>%
  dplyr::mutate(thresholded_annotation_type = case_when(
    
    #neither
    annotation_type == "neither" ~ "neither",
    annotation_type == "calico only" & calico_hyperGeomScore < optimal_calico_score_threshold ~ "neither",
    annotation_type == "msdial only" & msdial_hyperGeomScore < optimal_msdial_score_threshold ~ "neither",
    annotation_type == "both" & calico_hyperGeomScore < optimal_calico_score_threshold & msdial_hyperGeomScore < optimal_msdial_score_threshold ~ "neither",
    
    # calico only
    annotation_type == "calico only" & calico_hyperGeomScore >= optimal_calico_score_threshold ~ "calico only",
    annotation_type == "both" & calico_hyperGeomScore >= optimal_calico_score_threshold & msdial_hyperGeomScore < optimal_msdial_score_threshold ~ "calico only",
    
    # msdial only
    annotation_type == "msdial only" & msdial_hyperGeomScore >= optimal_msdial_score_threshold ~ "msdial only",
    annotation_type == "both" & calico_hyperGeomScore < optimal_calico_score_threshold & msdial_hyperGeomScore >= optimal_msdial_score_threshold ~ "msdial only",
    
    # both
    annotation_type == "both" & calico_hyperGeomScore >= optimal_calico_score_threshold & msdial_hyperGeomScore >= optimal_msdial_score_threshold ~ "both",
    TRUE==TRUE ~ "unhandled"
  ))
```

## Score summarized computation

```
scores_summarized_by_class = all_scores_results_thresholded %>%
  dplyr::filter(thresholded_annotation_type == "msdial only" | thresholded_annotation_type == "calico only" |
                (thresholded_annotation_type == "both" & is_agreement_class)) %>%
  dplyr::mutate(lipid_class = ifelse(thresholded_annotation_type == "msdial only", msdial_lipidClass_adj, calico_lipidClass)) %>%
  dplyr::group_by(thresholded_annotation_type, dataset, mode, lipid_class) %>%
  dplyr::mutate(class_count = n()) %>%
  dplyr::ungroup() %>%
  dplyr::select(thresholded_annotation_type, dataset, mode, lipid_class, class_count) %>%
  unique()
```

# Explore Full Disagreements Analysis

```
all_different <- all_scores_results_thresholded %>%
  dplyr::filter(thresholded_annotation_type == "both") %>%
  dplyr::filter(!is_agreement_class & !is_agreement_sumComposition & !is_agreement_compound & !is_agreement_adduct)
```

## Full Disagreement 1: (744.5516, 22.509840)

```
difference_best_calico <- all_different %>% 
  dplyr::filter(medMz > 744.55 & medMz < 744.56) %>%
  dplyr::select(medMz, medRt, dataset, mode, calico_compound_adj, msdial_compound_adj)

cat(paste0("(", round(difference_best_calico$medMz[1], digits=2), ", ", round(difference_best_calico$medRt[1], digits = 2), "): ",
           "<", difference_best_calico$dataset[1], " ", difference_best_calico$mode[1], "> ",
           difference_best_calico$calico_compound_adj[1]," <--> ", difference_best_calico$msdial_compound_adj[1],
           "\n"))
#> (744.55, 22.51): <thermo negative ionization mode> PC(16:0/18:1) <--> PE(16:0/20:1)
```

Calico Identification

MS-DIAL Identification

## Full Disagreement 2: (836.5784, 19.865680)

```
difference_best_msdial <- all_different %>% dplyr::filter(medMz > 836.57 & medMz < 836.58)

cat(paste0("(", round(difference_best_msdial$medMz[1], digits=2), ", ", round(difference_best_msdial$medRt[1], digits = 2), "): ",
           "<", difference_best_msdial$dataset[1], " ", difference_best_msdial$mode[1], "> ",
           difference_best_msdial$calico_compound_adj[1]," <--> ", difference_best_msdial$msdial_compound_adj[1],
           "\n"))
#> (836.58, 19.87): <thermo negative ionization mode> PE(20:1e/20:5) <--> PC(18:1e/20:5)
```

Calico Identification

MS-DIAL Identification

# Library Contents Analysis

## Library Reformatting

```
msdial_neg_compounds <- msdial_neg %>%
  dplyr::select(compoundName, adductName, lipidClass) %>%
  dplyr::rename(compound = compoundName, msdial_adduct = adductName) %>%
  unique()

msdial_pos_compounds <- msdial_pos %>%
  dplyr::select(compoundName, adductName, lipidClass) %>%
  dplyr::rename(compound = compoundName, msdial_adduct = adductName) %>%
  unique()

msdial_neg_compounds_reformatted <- msdial_compound_info(msdial_neg_compounds)
msdial_pos_compounds_reformatted <- msdial_compound_info(msdial_pos_compounds)

# Test to ensure that parsing did not inadvertently produce any NA values
cat(paste0("TEST: Negative mode MS-DIAL compounds correctly parsed? ", length(which(is.na(msdial_neg_compounds_reformatted)))==0, "\n"))
#> TEST: Negative mode MS-DIAL compounds correctly parsed? TRUE
cat(paste0("TEST: Positive mode MS-DIAL compounds correctly parsed? ", length(which(is.na(msdial_pos_compounds_reformatted)))==0, "\n"))
#> TEST: Positive mode MS-DIAL compounds correctly parsed? TRUE

# MS-DIAL neg library, in calico format
msdial_neg_lipid_components <- lipid_components(msdial_neg_compounds_reformatted$msdial_compound_adj %>% unique()) %>%
  dplyr::select(compoundName, etherPlasmalogenCompoundName, etherPlasmalogenSumComposition)

msdial_neg_compounds_complete <- dplyr::inner_join(msdial_neg_compounds_reformatted, msdial_neg_lipid_components,
                                                   by = c("msdial_compound_adj"="compoundName")) %>%
    dplyr::rename(msdial_sumComposition_adj = etherPlasmalogenSumComposition) %>%
    dplyr::select(-msdial_compound_adj) %>%
    dplyr::rename(msdial_compound_adj = etherPlasmalogenCompoundName) %>%
  dplyr::select(msdial_lipidClass_adj, msdial_sumComposition_adj, msdial_compound_adj, msdial_adduct)

# MS-DIAL pos library, in calico format
msdial_pos_lipid_components <- lipid_components(msdial_pos_compounds_reformatted$msdial_compound_adj %>% unique()) %>%
  dplyr::select(compoundName, etherPlasmalogenCompoundName, etherPlasmalogenSumComposition)

msdial_pos_compounds_complete <- dplyr::inner_join(msdial_pos_compounds_reformatted, msdial_pos_lipid_components,
                                                   by = c("msdial_compound_adj"="compoundName")) %>%
    dplyr::rename(msdial_sumComposition_adj = etherPlasmalogenSumComposition) %>%
    dplyr::select(-msdial_compound_adj) %>%
    dplyr::rename(msdial_compound_adj = etherPlasmalogenCompoundName) %>%
  dplyr::select(msdial_lipidClass_adj, msdial_sumComposition_adj, msdial_compound_adj, msdial_adduct)
  
msdial_combined_library <- rbind(msdial_neg_compounds_complete, msdial_pos_compounds_complete)

# Calico neg library
calico_neg_lipid_components <- lipid_components(calico_neg$compoundName %>% unique()) %>%
    dplyr::select(compoundName, etherPlasmalogenCompoundName, etherPlasmalogenSumComposition)

calico_neg_ms1 <- calico_neg %>%
  dplyr::select(lipidClass, compositionSummary, compoundName, adductName) %>%
  unique()

calico_neg_all <- dplyr::inner_join(calico_neg_ms1, calico_neg_lipid_components, by = c("compoundName")) %>%
    dplyr::rename(calico_sumComposition = etherPlasmalogenSumComposition) %>%
    dplyr::rename(calico_compound = etherPlasmalogenCompoundName) %>%
    
    # Avoid 0-length chain acyl chains for agreement with MS-DIAL libraries
    dplyr::mutate(calico_compound_adj = ifelse(lipidClass %in% c("LPC","LPE"), calico_sumComposition, calico_compound)) %>%
    
    dplyr::mutate(calico_compound_adj = ifelse(calico_compound_adj == "", "unidentified", calico_compound_adj)) %>%
    dplyr::rename(calico_adduct = adductName, calico_lipidClass = lipidClass) %>%
  dplyr::select(calico_lipidClass, calico_sumComposition, calico_compound_adj, calico_adduct)

# Calico pos library
calico_pos_lipid_components <- lipid_components(calico_pos$compoundName %>% unique()) %>%
    dplyr::select(compoundName, etherPlasmalogenCompoundName, etherPlasmalogenSumComposition)

calico_pos_ms1 <- calico_pos %>%
  dplyr::select(lipidClass, compositionSummary, compoundName, adductName) %>%
  unique()

calico_pos_all <- dplyr::inner_join(calico_pos_ms1, calico_pos_lipid_components, by = c("compoundName")) %>%
    dplyr::rename(calico_sumComposition = etherPlasmalogenSumComposition) %>%
    dplyr::rename(calico_compound = etherPlasmalogenCompoundName) %>%
    
    # Avoid 0-length chain acyl chains for agreement with MS-DIAL libraries
    dplyr::mutate(calico_compound_adj = ifelse(lipidClass %in% c("LPC","LPE"), calico_sumComposition, calico_compound)) %>%
    
    dplyr::mutate(calico_compound_adj = ifelse(calico_compound_adj == "", "unidentified", calico_compound_adj)) %>%
    dplyr::rename(calico_adduct = adductName, calico_lipidClass = lipidClass) %>%
  dplyr::select(calico_lipidClass, calico_sumComposition, calico_compound_adj, calico_adduct)

calico_combined_library <- rbind(calico_neg_all, calico_pos_all)
```

## Library Analysis

```
msdial_class_adduct_counts <- msdial_combined_library %>%
  dplyr::group_by(msdial_lipidClass_adj, msdial_adduct) %>%
  dplyr::mutate(msdial_count = n()) %>%
  dplyr::ungroup() %>%
  dplyr::select(msdial_lipidClass_adj, msdial_adduct, msdial_count) %>%
  unique()

calico_class_adduct_counts <- calico_combined_library %>%
  dplyr::group_by(calico_lipidClass, calico_adduct) %>%
  dplyr::mutate(calico_count = n()) %>%
  dplyr::ungroup() %>%
  dplyr::select(calico_lipidClass, calico_adduct, calico_count) %>%
  unique()

msdial_id_counts <- all_scores_results_thresholded %>%
  dplyr::filter(msdial_hyperGeomScore >= optimal_msdial_score_threshold) %>%
  dplyr::group_by(msdial_lipidClass_adj, msdial_adduct) %>%
  dplyr::mutate(msdial_id_count = n()) %>%
  dplyr::ungroup() %>%
  dplyr::select(msdial_lipidClass_adj, msdial_adduct, msdial_id_count) %>%
  unique()

calico_id_counts <- all_scores_results_thresholded %>%
  dplyr::filter(calico_hyperGeomScore >= optimal_calico_score_threshold) %>%
  dplyr::group_by(calico_lipidClass, calico_adduct) %>%
  dplyr::mutate(calico_id_count = n()) %>%
  dplyr::ungroup() %>%
  dplyr::select(calico_lipidClass, calico_adduct, calico_id_count) %>%
  unique()

msdial_all_counts <- dplyr::full_join(msdial_class_adduct_counts, msdial_id_counts, by = c("msdial_lipidClass_adj","msdial_adduct")) %>%
  dplyr::mutate(msdial_id_count = ifelse(is.na(msdial_id_count), 0, msdial_id_count)) %>%
  dplyr::arrange(msdial_lipidClass_adj, msdial_adduct)

calico_all_counts <- dplyr::full_join(calico_class_adduct_counts, calico_id_counts, by = c("calico_lipidClass","calico_adduct")) %>%
  dplyr::mutate(calico_id_count = ifelse(is.na(calico_id_count), 0, calico_id_count)) %>%
  dplyr::arrange(calico_lipidClass, calico_adduct)

both_libs <- dplyr::inner_join(calico_all_counts, msdial_all_counts, by = c("calico_lipidClass"="msdial_lipidClass_adj","calico_adduct"="msdial_adduct")) %>%
  dplyr::rename(`Lipid Class` = calico_lipidClass, Adduct = calico_adduct)

both_libs_identified <- dplyr::inner_join(calico_all_counts, msdial_all_counts, by = c("calico_lipidClass"="msdial_lipidClass_adj","calico_adduct"="msdial_adduct")) %>%
  dplyr::rename(`Lipid Class` = calico_lipidClass, Adduct = calico_adduct) %>%
  dplyr::filter(calico_id_count > 0 | msdial_id_count > 0)

calico_only_libs <- dplyr::anti_join(calico_all_counts, msdial_all_counts, by = c("calico_lipidClass"="msdial_lipidClass_adj","calico_adduct"="msdial_adduct")) %>%
  dplyr::rename(`Lipid Class` = calico_lipidClass, Adduct = calico_adduct) %>%
  dplyr::filter(calico_id_count > 0)

msdial_only_libs <- dplyr::anti_join(msdial_all_counts, calico_all_counts, by = c("msdial_lipidClass_adj"="calico_lipidClass","msdial_adduct"="calico_adduct")) %>%
  dplyr::rename(`Lipid Class` = msdial_lipidClass_adj, Adduct = msdial_adduct) %>%
  dplyr::filter(msdial_id_count > 0)
```

# Visualizations

## Agreement Score Distributions

```
print(plot_dist(all_scores_results_class_agreement, plot_title="Class Agreement"))
```

```
print(plot_dist(all_scores_results_class_and_adduct_agreement, plot_title="Class + Adduct Agreement"))
```

```
print(plot_dist(all_scores_results_composition_agreement, plot_title="Composition Agreement"))
```

```
print(plot_dist(all_scores_results_compound_agreement, plot_title="Compound Agreement"))
```

## Agreement and Disagreement Distributions

```
b_width <- 0.3
agreements_plot <- ggplot(agreements_and_disagreements, aes(score, fill = type)) +
    #geom_density(alpha=0.5)  
    geom_histogram(binwidth = b_width, alpha = 0.5, position="dodge") +
    ggtitle("All Agreements and Disagreements")

# print(agreements_plot)

calico_agreements_plot <- ggplot(calico_AD, aes(score, fill = type)) +
    #geom_density(alpha=0.5)  
    geom_histogram(binwidth = 0.6, alpha = 0.5, position="dodge") +
    ggtitle("Calico Agreements and Disagreements")

print(calico_agreements_plot)
```

```
msdial_agreements_plot <- ggplot(msdial_AD, aes(score, fill = type)) +
    #geom_density(alpha=0.5)  
    geom_histogram(binwidth = b_width, alpha = 0.5, position="dodge") +
    ggtitle("MS-DIAL Agreements and Disagreements")

print(msdial_agreements_plot)
```

## Agreements and Disagreements Together

```
b_width <- 1
AD_together_plot <- ggplot(AD_together, aes(score, fill = Type)) +
  facet_wrap(~Library, ncol =1) +
  geom_histogram(binwidth = b_width, alpha = 0.5, position="dodge") +
  ggtitle("Lipid Annotation Agreements and Disagreements by Library and Score") +
  ylab("Number of Annotated Features") +
  xlab("Hypergeometric Score")

print(AD_together_plot)
```

## Print score threshold plot

```
plot_score_thresholds_raw <- ggplot(both_series, aes(x=score)) +
  geom_point(aes(y=calico_fraction_diff, colour="calico"), show.legend=TRUE) +
  geom_point(aes(y=msdial_fraction_diff, colour="msdial"), show.legend=TRUE) +
  xlab("Hypergeometric Score") +
  ylab("(Fraction of agreements retained - Fraction of disagreements retained)") +
  ggtitle("Agreement and Disagreement Retention (raw data)") +
  labs(colour="library")

print(plot_score_thresholds_raw)
```

```
plot_score_thresholds <- ggplot(both_series, aes(x=score)) +
  geom_smooth(aes(y=calico_fraction_diff, color="calico"), show.legend=TRUE) +
  geom_smooth(aes(y=msdial_fraction_diff, color="msdial"), show.legend=TRUE) +
  xlab("Hypergeometric Score") +
  ylab("(Fraction of agreements retained - Fraction of disagreements retained)") +
  geom_vline(xintercept = optimal_calico_score_threshold) +
  annotate(geom="vline",
          xintercept=optimal_calico_score_threshold) +
  annotate(geom="text",
             label=paste0(" score threshold=",optimal_calico_score_threshold),
             x=optimal_calico_score_threshold,
             y=0.6,
             hjust=0) +
    geom_vline(xintercept = optimal_msdial_score_threshold) +
    annotate(geom="vline",
             xintercept=optimal_msdial_score_threshold) +
    annotate(geom="text",
             label=paste0("score threshold=",optimal_msdial_score_threshold, " "),
             x=optimal_msdial_score_threshold,
             y=0.7,
             hjust=1) +
  ggtitle("Agreement and Disagreement Retention (Fit)") +
  labs(colour="library")

print(plot_score_thresholds)
#> `geom_smooth()` using method = 'gam' and formula 'y ~ s(x, bs = "cs")'
#> `geom_smooth()` using method = 'gam' and formula 'y ~ s(x, bs = "cs")'
```

## Figure 4: Agreement and Disagreement Retention Plot

```
library_colors <- c(calico="#E41A1C", msdial="#377EB8")

plot_all_no_annotations <- ggplot(both_series, aes(x=score)) +
  
  theme_bw(base_size = 14) +
  
  geom_point(aes(y=calico_fraction_diff, shape="calico"), show.legend=TRUE) +
  geom_point(aes(y=msdial_fraction_diff, shape="msdial"), show.legend=TRUE) +
  
  geom_smooth(aes(y=calico_fraction_diff, colour="calico"), show.legend=TRUE) +
  geom_smooth(aes(y=msdial_fraction_diff, colour="msdial"), show.legend=TRUE) +
  
  #ggtitle("Agreement and Disagreement Retention Fraction (Raw Data + Fit)") +
  theme(plot.title = element_text(hjust = 0.5)) +
  xlab("Hypergeometric Score") +
  ylab("Retention Fraction Difference") +
  
  labs(colour="library", shape="library") +

  scale_colour_discrete(
                        type=library_colors,
                        name="Fit",
                        breaks=c("calico","msdial"),
                        labels=c("Calico","MS-DIAL"),
                        guide = guide_legend(override.aes =
                                               list(shape = NA, fill=NA, colour = c("#E41A1C","#377EB8"))
                                             )
                        ) +
  scale_shape_discrete(name="Data Source", breaks=c("calico","msdial"), labels=c("Calico", "MS-DIAL"),
                       guide = guide_legend(override.aes = 
                                              list(linetype= c(0,0), colour="black", fill=NA)
                                            )
                       )
  
plot_all_2col <- plot_all_no_annotations +
  geom_vline(xintercept = optimal_calico_score_threshold) +
  annotate(geom="vline",
          xintercept=optimal_calico_score_threshold) +
  annotate(geom="text",
             label=paste0(" RBP=",optimal_calico_score_threshold),
             x=optimal_calico_score_threshold,
             y=0.6,
             hjust=0) +
    geom_vline(xintercept = optimal_msdial_score_threshold) +
    annotate(geom="vline",
             xintercept=optimal_msdial_score_threshold) +
    annotate(geom="text",
             label=paste0("RBP=",optimal_msdial_score_threshold, " "),
             x=optimal_msdial_score_threshold,
             y=0.7,
             hjust=1)

print(plot_all_2col)
```

## Optimal Score Threshold Plots

```
calico_agreements_plot_w_score_thresh <- ggplot(calico_AD, aes(score, fill = type)) +
    geom_histogram(binwidth = 0.6, alpha = 0.5, position="dodge") +
    ggtitle("Calico Agreements and Disagreements") +
    geom_vline(xintercept = optimal_calico_score_threshold) +
    annotate(geom="vline",
             xintercept=optimal_calico_score_threshold) +
    annotate(geom="text",
             label=paste0(" score threshold=",optimal_calico_score_threshold),
             x=optimal_calico_score_threshold,
             y=70,
             hjust=0)

print(calico_agreements_plot_w_score_thresh)
```

```
msdial_agreements_plot_w_score_thresh <- ggplot(msdial_AD, aes(score, fill = type)) +
    geom_histogram(binwidth = b_width, alpha = 0.5, position="dodge") +
    ggtitle("MS-DIAL Agreements and Disagreements") +
    geom_vline(xintercept = optimal_msdial_score_threshold) +
    annotate(geom="vline",
             xintercept=optimal_msdial_score_threshold) +
    annotate(geom="text",
             label=paste0(" score threshold=",optimal_msdial_score_threshold),
             x=optimal_msdial_score_threshold,
             y=87.5,
             hjust=0)
print(msdial_agreements_plot_w_score_thresh)
```

## Table 1: Lipid Annotation Agreement

```
## FEATURES

num_neither <- all_scores_results_thresholded %>%
  dplyr::filter(thresholded_annotation_type == "neither") %>%
  nrow()

num_annotated <- all_scores_results_thresholded %>%
  dplyr::filter(thresholded_annotation_type != "neither") %>%
  nrow()

num_calico_only <- all_scores_results_thresholded %>%
  dplyr::filter(thresholded_annotation_type == "calico only") %>%
  nrow()

num_msdial_only <- all_scores_results_thresholded %>%
  dplyr::filter(thresholded_annotation_type == "msdial only") %>%
  nrow()

num_both <- all_scores_results_thresholded %>%
  dplyr::filter(thresholded_annotation_type == "both") %>%
  nrow()

num_both_same_class <- all_scores_results_thresholded %>%
  dplyr::filter(thresholded_annotation_type == "both") %>%
  dplyr::filter(is_agreement_class) %>%
  nrow()

num_both_same_class_and_adduct <- all_scores_results_thresholded %>%
  dplyr::filter(thresholded_annotation_type == "both") %>%
  dplyr::filter(is_agreement_class & is_agreement_adduct) %>%
  nrow()

num_both_same_sum_composition <- all_scores_results_thresholded %>%
  dplyr::filter(thresholded_annotation_type == "both") %>%
  dplyr::filter(is_agreement_class & is_agreement_sumComposition & is_agreement_adduct) %>%
  nrow()

num_both_same_compound <- all_scores_results_thresholded %>%
  dplyr::filter(thresholded_annotation_type == "both") %>%
  dplyr::filter(is_agreement_class & is_agreement_sumComposition & is_agreement_compound & is_agreement_adduct) %>%
  nrow()

num_both_all_different <- all_scores_results_thresholded %>%
  dplyr::filter(thresholded_annotation_type == "both") %>%
  dplyr::filter(!is_agreement_class & !is_agreement_sumComposition & !is_agreement_compound & !is_agreement_adduct) %>%
  nrow()

## COMPOUND COUNTS

numc_calico_only <- all_scores_results_thresholded %>%
  dplyr::filter(thresholded_annotation_type == "calico only") %>%
  dplyr::select(calico_compound_adj) %>%
  unique() %>%
  nrow()

numc_msdial_only <- all_scores_results_thresholded %>%
  dplyr::filter(thresholded_annotation_type == "msdial only") %>%
  dplyr::select(msdial_compound_adj) %>%
  unique() %>%
  nrow()

numc_both_same_compound <- all_scores_results_thresholded %>%
  dplyr::filter(thresholded_annotation_type == "both") %>%
  dplyr::filter(is_agreement_class & is_agreement_sumComposition & is_agreement_compound & is_agreement_adduct) %>%
  dplyr::select(calico_compound_adj) %>%
  unique() %>%
  nrow()

## VECTORS

description <- c(
  "All Features",
  "Unannotated",
  "Annotated",
  "Identified only in Calico library",
  "Identified only in MS-DIAL library",
  "Identified by both libraries",
  "Identified by both libraries with same lipid class",
  "Identified by both libraries with same lipid class and adduct",
  "Identified by both libraries with same summed composition and adduct",
  "Identified by both libraries as same compound (full agreement)",
  "Identified by both libraries with different lipid class, adduct, summed composition and compound (full disagreement)"
  )

features <- c(
  all_scores_results_thresholded %>% nrow(),
  num_neither,
  num_annotated,
  num_calico_only,
  num_msdial_only,
  num_both,
  num_both_same_class,
  num_both_same_class_and_adduct,
  num_both_same_sum_composition,
  num_both_same_compound,
  num_both_all_different
)

compounds <- c(
  "--",
  "--",
  "--",
  as.character(numc_calico_only),
  as.character(numc_msdial_only),
  "--",
  "--",
  "--",
  "--",
  as.character(numc_both_same_compound),
  "--"
)

pct_features <- round(100 * (features / (all_scores_results_thresholded %>% nrow())),digits=1)

ann_results_table <- tibble::tibble(
  "Annotation Type" = description,
  "# Features" = features,
  "% Features" = pct_features,
  "# Compounds" = compounds
  )

ann_results_table <- ann_results_table %>%
  dplyr::mutate(`% Features` = paste0(`% Features`, "%"))

knitr::kable(ann_results_table, "html") %>%
  kableExtra::kable_styling(bootstrap_options = c("striped", "hover", "responsive"), position = "left", full_width = FALSE) %>%
  kableExtra::row_spec(1:nrow(ann_results_table), bold = T, color = "#CCCCCC", background = "#990066",font_size = "xx-large")
```

| Annotation Type | # Features | % Features | # Compounds |
| --- | --- | --- | --- |
| All Features | 12054 | 100% | -- |
| Unannotated | 9442 | 78.3% | -- |
| Annotated | 2612 | 21.7% | -- |
| Identified only in Calico library | 1009 | 8.4% | 768 |
| Identified only in MS-DIAL library | 461 | 3.8% | 429 |
| Identified by both libraries | 1142 | 9.5% | -- |
| Identified by both libraries with same lipid class | 1078 | 8.9% | -- |
| Identified by both libraries with same lipid class and adduct | 994 | 8.2% | -- |
| Identified by both libraries with same summed composition and adduct | 990 | 8.2% | -- |
| Identified by both libraries as same compound (full agreement) | 512 | 4.2% | 253 |
| Identified by both libraries with different lipid class, adduct, summed composition and compound (full disagreement) | 44 | 0.4% | -- |

## Table 2: Feature Identification

```
instrument <- c(rep(c("Internal Thermo Q Exactive Dataset"), 4),rep(c("External Agilent 6546 QTOF Dataset"), 4))
library_name <- rep(c("Calico","MS-DIAL"),4)
mode <- rep(c(rep(c("positive"), 2), rep(c("negative"), 2)), 2)

num_IDs <- c(
  all_scores_results_thresholded %>% dplyr::filter(
    dataset == "thermo" & mode == "positive ionization mode" & (thresholded_annotation_type == "both" | thresholded_annotation_type == "calico only")) %>%
    nrow(),
  all_scores_results_thresholded %>% dplyr::filter(
    dataset == "thermo" & mode == "positive ionization mode" & (thresholded_annotation_type == "both" | thresholded_annotation_type == "msdial only")) %>%
    nrow(),
  all_scores_results_thresholded %>% dplyr::filter(
    dataset == "thermo" & mode == "negative ionization mode" & (thresholded_annotation_type == "both" | thresholded_annotation_type == "calico only")) %>%
    nrow(),
  all_scores_results_thresholded %>% dplyr::filter(
    dataset == "thermo" & mode == "negative ionization mode" & (thresholded_annotation_type == "both" | thresholded_annotation_type == "msdial only")) %>%
    nrow(),
  all_scores_results_thresholded %>% dplyr::filter(
    dataset == "agilent" & mode == "positive ionization mode" & (thresholded_annotation_type == "both" | thresholded_annotation_type == "calico only")) %>%
    nrow(),
  all_scores_results_thresholded %>% dplyr::filter(
    dataset == "agilent" & mode == "positive ionization mode" & (thresholded_annotation_type == "both" | thresholded_annotation_type == "msdial only")) %>%
    nrow(),
  all_scores_results_thresholded %>% dplyr::filter(
    dataset == "agilent" & mode == "negative ionization mode" & (thresholded_annotation_type == "both" | thresholded_annotation_type == "calico only")) %>%
    nrow(),
  all_scores_results_thresholded %>% dplyr::filter(
    dataset == "agilent" & mode == "negative ionization mode" & (thresholded_annotation_type == "both" | thresholded_annotation_type == "msdial only")) %>%
    nrow()
)

num_features <- c(
  thermo_calico_pos %>% nrow(),
  thermo_calico_pos %>% nrow(),
  thermo_calico_neg %>% nrow(),
  thermo_calico_neg %>% nrow(),
  agilent_calico_pos %>% nrow(),
  agilent_calico_pos %>% nrow(),
  agilent_calico_neg %>% nrow(),
  agilent_calico_neg %>% nrow()
)

num_compounds <- c(
   all_scores_results_thresholded %>% dplyr::filter(
     dataset == "thermo" & mode == "positive ionization mode" & (thresholded_annotation_type == "both" | thresholded_annotation_type == "calico only")) %>%
     dplyr::select(calico_compound_adj) %>%
     unique() %>%
     nrow(),
    all_scores_results_thresholded %>% dplyr::filter(
     dataset == "thermo" & mode == "positive ionization mode" & (thresholded_annotation_type == "both" | thresholded_annotation_type == "msdial only")) %>%
     dplyr::select(msdial_compound_adj) %>%
     unique() %>%
     nrow(),
   all_scores_results_thresholded %>% dplyr::filter(
     dataset == "thermo" & mode == "negative ionization mode" & (thresholded_annotation_type == "both" | thresholded_annotation_type == "calico only")) %>%
     dplyr::select(calico_compound_adj) %>%
     unique() %>%
     nrow(),
   all_scores_results_thresholded %>% dplyr::filter(
     dataset == "thermo" & mode == "negative ionization mode" & (thresholded_annotation_type == "both" | thresholded_annotation_type == "msdial only")) %>%
     dplyr::select(msdial_compound_adj) %>%
     unique() %>%
     nrow(),
   all_scores_results_thresholded %>% dplyr::filter(
     dataset == "agilent" & mode == "positive ionization mode" & (thresholded_annotation_type == "both" | thresholded_annotation_type == "calico only")) %>%
     dplyr::select(calico_compound_adj) %>%
     unique() %>%
     nrow(),
   all_scores_results_thresholded %>% dplyr::filter(
     dataset == "agilent" & mode == "positive ionization mode" & (thresholded_annotation_type == "both" | thresholded_annotation_type == "msdial only")) %>%
     dplyr::select(msdial_compound_adj) %>%
     unique() %>%
     nrow(),
   all_scores_results_thresholded %>% dplyr::filter(
     dataset == "agilent" & mode == "negative ionization mode" & (thresholded_annotation_type == "both" | thresholded_annotation_type == "calico only")) %>%
     dplyr::select(calico_compound_adj) %>%
     unique() %>%
     nrow(),
  all_scores_results_thresholded %>% dplyr::filter(
    dataset == "agilent" & mode == "negative ionization mode" & (thresholded_annotation_type == "both" | thresholded_annotation_type == "msdial only")) %>%
    dplyr::select(msdial_compound_adj) %>%
    unique() %>%
    nrow()
)

percent_IDS <- 100 * num_IDs / num_features
percent_IDS_chr <- as.character(round(percent_IDS, digits=1))

results_table <- tibble::tibble(
  "Dataset" = instrument,
  "Library" = library_name,
  "Ionization Mode" = mode,
  "# Features" = num_features,
  "# Features Identified" = num_IDs,
  "% Features Identified" = percent_IDS_chr,
  "# Compounds Identified" = num_compounds
  )

results_table <- results_table %>%
  dplyr::mutate(`% Features Identified` = paste0(`% Features Identified`, "%"))

knitr::kable(results_table, "html") %>%
  kableExtra::kable_styling(bootstrap_options = c("striped", "hover", "responsive"), position = "left", full_width = FALSE) %>%
  kableExtra::row_spec(1:nrow(results_table), bold = T, color = "#CCCCCC", background = "#990066",font_size = "xx-large")
```

| Dataset | Library | Ionization Mode | # Features | # Features Identified | % Features Identified | # Compounds Identified |
| --- | --- | --- | --- | --- | --- | --- |
| Internal Thermo Q Exactive Dataset | Calico | positive | 3412 | 995 | 29.2% | 741 |
| Internal Thermo Q Exactive Dataset | MS-DIAL | positive | 3412 | 525 | 15.4% | 442 |
| Internal Thermo Q Exactive Dataset | Calico | negative | 1301 | 384 | 29.5% | 300 |
| Internal Thermo Q Exactive Dataset | MS-DIAL | negative | 1301 | 304 | 23.4% | 263 |
| External Agilent 6546 QTOF Dataset | Calico | positive | 4162 | 502 | 12.1% | 415 |
| External Agilent 6546 QTOF Dataset | MS-DIAL | positive | 4162 | 378 | 9.1% | 358 |
| External Agilent 6546 QTOF Dataset | Calico | negative | 3179 | 270 | 8.5% | 245 |
| External Agilent 6546 QTOF Dataset | MS-DIAL | negative | 3179 | 396 | 12.5% | 362 |

## Figure 5: ID by class plot

```
scores_clean <- scores_summarized_by_class %>%
  dplyr::rename(Library = thresholded_annotation_type) %>%
  dplyr::mutate(dataset = case_when(
    dataset == "agilent" ~ "External Agilent 6546 QTOF Dataset",
    dataset == "thermo" ~ "Internal Thermo Q Exactive Dataset"
  )) %>%
  dplyr::mutate(Library = case_when(
    Library == "both" ~ "Both",
    Library == "calico only" ~ "Calico",
    Library == "msdial only" ~ "MS-DIAL"
  ))

#class_min <- 20
top_10_classes <- c("Alkyl_PC","Alkyl_PE","Ceramide","LPC","PC","PE","PI","SM","TG")

dataset_factor <- factor(scores_clean$dataset, levels=c("Internal Thermo Q Exactive Dataset", "External Agilent 6546 QTOF Dataset"))

scores_clean_factors <- scores_clean %>% 
  dplyr::mutate(dataset = dataset_factor) %>%
  dplyr::mutate(lumped_class = ifelse(lipid_class %in% top_10_classes, lipid_class, "other")) %>%
  dplyr::group_by(Library, dataset, lumped_class) %>%
  dplyr::mutate(lumped_class_count = sum(class_count)) %>%
  dplyr::ungroup()

lumped_class_factor <- factor(scores_clean_factors$lumped_class, levels=c(top_10_classes, "other"))
  
scores_lumped <- scores_clean_factors %>%
  dplyr::select(Library, dataset, lumped_class, lumped_class_count) %>%
  dplyr::mutate(lumped_class = lumped_class_factor) %>%
  unique()

p_id_by_class <- ggplot(data = scores_lumped, aes(x = lumped_class, y = lumped_class_count, fill = Library)) +
  
  #geom_bar(stat="identity") +
  geom_bar(stat="identity", position=position_dodge()) +
  
  facet_wrap(~dataset, ncol =1) +
  theme(axis.text.x = element_text(size = 8, angle = 65, vjust = 1, hjust = 1)) +
  #theme(axis.text.x = element_blank()) +
  #labs(title = "Annotated Features by Lipid Class") +
  #theme(plot.title = element_text(hjust = 0.5)) +
  xlab("Lipid Class") +
  ylab("# Annotated Features") +
  theme_bw(base_size = 14) +
  theme(axis.text.x = element_text(angle=45, hjust=1)) +
  scale_fill_brewer(palette="Set1") +
  theme(strip.background = element_rect(fill="white"))

print(p_id_by_class)
```

## Calico Library Contents

```
knitr::kable(calico_all_counts, "html") %>%
  kableExtra::kable_styling(bootstrap_options = c("striped", "hover", "responsive"), position = "left", full_width = FALSE) %>%
  kableExtra::row_spec(1:nrow(calico_all_counts), bold = T, color = "#CCCCCC", background = "#990066",font_size = "xx-large")
```

| calico\_lipidClass | calico\_adduct | calico\_count | calico\_id\_count |
| --- | --- | --- | --- |
| AcGD1a | [M-2H]2- | 9072 | 0 |
| AcGD1a | [M-H]- | 9072 | 0 |
| AcGD1a | [M+2H]2+ | 9072 | 0 |
| AcGD1a | [M+H]+ | 9072 | 0 |
| AcGD1a | [M+K]+ | 9072 | 0 |
| AcGD1a | [M+Na]+ | 9072 | 0 |
| AcGD1b | [M-2H]2- | 9072 | 0 |
| AcGD1b | [M-H]- | 9072 | 0 |
| AcGD1b | [M+2H]2+ | 9072 | 0 |
| AcGD1b | [M+H]+ | 9072 | 0 |
| AcGD1b | [M+K]+ | 9072 | 0 |
| AcGD1b | [M+Na]+ | 9072 | 0 |
| AcGD2 | [M-2H]2- | 9072 | 0 |
| AcGD2 | [M-H]- | 9072 | 0 |
| AcGD2 | [M+H]+ | 9072 | 0 |
| AcGD2 | [M+Na]+ | 9072 | 0 |
| AcGD3 | [M-2H]2- | 9072 | 0 |
| AcGD3 | [M-H]- | 9072 | 0 |
| AcGD3 | [M+H]+ | 9072 | 0 |
| AcGM1 | [M-H]- | 9072 | 0 |
| AcGM1 | [M+H]+ | 9072 | 0 |
| AcGM2 | [M-H]- | 9072 | 0 |
| AcGM2 | [M+H]+ | 9072 | 0 |
| AcGM3 | [M-H]- | 9072 | 1 |
| AcGM3 | [M+H]+ | 9072 | 2 |
| AcGQ1b | [M-2H]2- | 9072 | 0 |
| AcGQ1b | [M-3H]3- | 9072 | 0 |
| AcGQ1b | [M+2H]2+ | 9072 | 0 |
| AcGQ1b | [M+H]+ | 9072 | 0 |
| AcGT1b | [M-2H]2- | 9072 | 0 |
| AcGT1b | [M+2H]2+ | 9072 | 0 |
| AcGT1b | [M+H]+ | 9072 | 0 |
| AcGT1b | [M+Na]+ | 9072 | 0 |
| Alkyl\_LPC | [M-CH3]- | 39 | 0 |
| Alkyl\_LPC | [M-H]- | 39 | 0 |
| Alkyl\_LPC | [M+AcOH-H]- | 39 | 0 |
| Alkyl\_LPC | [M+Cl]- | 39 | 0 |
| Alkyl\_LPC | [M+FA-H]- | 39 | 0 |
| Alkyl\_LPC | [M+H]+ | 39 | 0 |
| Alkyl\_LPC | [M+K]+ | 39 | 0 |
| Alkyl\_LPC | [M+Na]+ | 39 | 0 |
| Alkyl\_LPC | [M+NH4]+ | 39 | 0 |
| Alkyl\_LPE | [M-H]- | 39 | 0 |
| Alkyl\_LPE | [M+AcOH-H]- | 39 | 0 |
| Alkyl\_LPE | [M+Cl]- | 39 | 0 |
| Alkyl\_LPE | [M+FA-H]- | 39 | 0 |
| Alkyl\_LPE | [M+H]+ | 39 | 0 |
| Alkyl\_LPE | [M+K]+ | 39 | 0 |
| Alkyl\_LPE | [M+Na]+ | 39 | 0 |
| Alkyl\_LPE | [M+NH4]+ | 39 | 0 |
| Alkyl\_LPS | [M-H]- | 39 | 0 |
| Alkyl\_LPS | [M+AcOH-H]- | 39 | 0 |
| Alkyl\_LPS | [M+Cl]- | 39 | 0 |
| Alkyl\_LPS | [M+FA-H]- | 39 | 0 |
| Alkyl\_PC | [M-CH3]- | 3627 | 0 |
| Alkyl\_PC | [M+AcOH-H]- | 3627 | 17 |
| Alkyl\_PC | [M+Cl]- | 3627 | 1 |
| Alkyl\_PC | [M+FA-H]- | 3627 | 20 |
| Alkyl\_PC | [M+H]+ | 3627 | 81 |
| Alkyl\_PC | [M+K]+ | 3627 | 1 |
| Alkyl\_PC | [M+Na]+ | 3627 | 20 |
| Alkyl\_PC | [M+NH4]+ | 3627 | 0 |
| Alkyl\_PE | [M-H]- | 3627 | 53 |
| Alkyl\_PE | [M+AcOH-H]- | 3627 | 1 |
| Alkyl\_PE | [M+Cl]- | 3627 | 0 |
| Alkyl\_PE | [M+FA-H]- | 3627 | 0 |
| Alkyl\_PE | [M+H]+ | 3627 | 31 |
| Alkyl\_PE | [M+K]+ | 3627 | 6 |
| Alkyl\_PE | [M+Na]+ | 3627 | 23 |
| Alkyl\_PE | [M+NH4]+ | 3627 | 0 |
| Alkyl\_PS | [M-H]- | 3627 | 0 |
| Alkyl\_PS | [M+AcOH-H]- | 3627 | 0 |
| Alkyl\_PS | [M+Cl]- | 3627 | 0 |
| Alkyl\_PS | [M+FA-H]- | 3627 | 0 |
| APCS | [M-H]- | 204 | 0 |
| APCS | [M+H]+ | 204 | 3 |
| BMP | [M-H]- | 8649 | 1 |
| BMP | [M+AcOH-H]- | 8649 | 0 |
| BMP | [M+Cl]- | 8649 | 0 |
| BMP | [M+FA-H]- | 8649 | 0 |
| BMP | [M+H]+ | 8649 | 0 |
| Carn | [M+H]+ | 204 | 23 |
| Carn | [M+K]+ | 204 | 0 |
| Carn | [M+Na]+ | 204 | 0 |
| Carn | [M+NH4]+ | 204 | 0 |
| CDP\_DG | [M-H]- | 8649 | 1 |
| CDP\_DG | [M+AcOH-H]- | 8649 | 0 |
| CDP\_DG | [M+Cl]- | 8649 | 0 |
| CDP\_DG | [M+FA-H]- | 8649 | 0 |
| CDP\_DG | [M+H]+ | 8649 | 0 |
| CDP\_DG | [M+K]+ | 8649 | 0 |
| CDP\_DG | [M+Na]+ | 8649 | 0 |
| CE | [M+K]+ | 204 | 0 |
| CE | [M+Na]+ | 204 | 4 |
| CE | [M+NH4]+ | 204 | 0 |
| Ceramide | [M-H]- | 9072 | 6 |
| Ceramide | [M+AcOH-H]- | 9072 | 31 |
| Ceramide | [M+Cl]- | 9072 | 0 |
| Ceramide | [M+FA-H]- | 9072 | 19 |
| Ceramide | [M+H]+ | 9072 | 34 |
| Ceramide | [M+K]+ | 9072 | 0 |
| Ceramide | [M+Na]+ | 9072 | 0 |
| Ceramide | [M+NH4]+ | 9072 | 0 |
| Ceramide\_P | [M-H]- | 9072 | 0 |
| Ceramide\_P | [M+AcOH-H]- | 9072 | 0 |
| Ceramide\_P | [M+Cl]- | 9072 | 0 |
| Ceramide\_P | [M+FA-H]- | 9072 | 0 |
| Ceramide\_P | [M+H]+ | 9072 | 0 |
| Ceramide\_P | [M+K]+ | 9072 | 0 |
| Ceramide\_P | [M+Na]+ | 9072 | 0 |
| Ceramide\_P | [M+NH4]+ | 9072 | 0 |
| CPE | [M-H]- | 9072 | 0 |
| CPE | [M+AcOH-H]- | 9072 | 0 |
| CPE | [M+Cl]- | 9072 | 0 |
| CPE | [M+FA-H]- | 9072 | 0 |
| CPE | [M+H]+ | 9072 | 2 |
| CPE | [M+K]+ | 9072 | 0 |
| CPE | [M+Na]+ | 9072 | 7 |
| CPE | [M+NH4]+ | 9072 | 0 |
| CPI | [M-H]- | 9072 | 3 |
| CPI | [M+AcOH-H]- | 9072 | 4 |
| CPI | [M+Cl]- | 9072 | 2 |
| CPI | [M+FA-H]- | 9072 | 0 |
| CPI | [M+H]+ | 9072 | 0 |
| CPI | [M+K]+ | 9072 | 0 |
| CPI | [M+Na]+ | 9072 | 0 |
| CPI | [M+NH4]+ | 9072 | 0 |
| DG | [M+K]+ | 8649 | 0 |
| DG | [M+Na]+ | 8649 | 10 |
| DG | [M+NH4]+ | 8649 | 0 |
| DGDG | [M-H]- | 8649 | 0 |
| DGDG | [M+AcOH-H]- | 8649 | 0 |
| DGDG | [M+Cl]- | 8649 | 0 |
| DGDG | [M+FA-H]- | 8649 | 0 |
| DGDG | [M+H]+ | 8649 | 0 |
| DGDG | [M+K]+ | 8649 | 0 |
| DGDG | [M+Na]+ | 8649 | 0 |
| DGDG | [M+NH4]+ | 8649 | 0 |
| DGTS | [M+H]+ | 8649 | 0 |
| DMPE | [M+H]+ | 8649 | 1 |
| DMPE | [M+K]+ | 8649 | 0 |
| DMPE | [M+Na]+ | 8649 | 0 |
| DMPE | [M+NH4]+ | 8649 | 0 |
| ErgE | [M+H]+ | 204 | 0 |
| ErgE | [M+K]+ | 204 | 0 |
| ErgE | [M+Na]+ | 204 | 0 |
| ErgE | [M+NH4]+ | 204 | 0 |
| Ethanolamine | [M+H]+ | 204 | 0 |
| Ethanolamine | [M+K]+ | 204 | 0 |
| Ethanolamine | [M+Na]+ | 204 | 0 |
| Ethanolamine | [M+NH4]+ | 204 | 0 |
| FA | [M-H]- | 204 | 0 |
| FA | [M+AcOH-H]- | 204 | 0 |
| FA | [M+Cl]- | 204 | 0 |
| FA | [M+FA-H]- | 204 | 0 |
| FAHFA | [M-H]- | 8649 | 2 |
| FAHFA | [M+AcOH-H]- | 8649 | 0 |
| FAHFA | [M+Cl]- | 8649 | 0 |
| FAHFA | [M+FA-H]- | 8649 | 0 |
| FAHFA | [M+K]+ | 8649 | 0 |
| FAHFA | [M+Na]+ | 8649 | 0 |
| FAHFA | [M+NH4]+ | 8649 | 0 |
| GB3 | [M-H]- | 9072 | 0 |
| GB3 | [M+AcOH-H]- | 9072 | 0 |
| GB3 | [M+Cl]- | 9072 | 0 |
| GB3 | [M+FA-H]- | 9072 | 0 |
| GB3 | [M+H]+ | 9072 | 2 |
| GB3 | [M+K]+ | 9072 | 0 |
| GB3 | [M+Na]+ | 9072 | 0 |
| GcGM2 | [M-H]- | 9072 | 7 |
| GcGM2 | [M+AcOH-H]- | 9072 | 0 |
| GcGM2 | [M+Cl]- | 9072 | 0 |
| GcGM2 | [M+FA-H]- | 9072 | 0 |
| GcGM2 | [M+H]+ | 9072 | 6 |
| GcGM2 | [M+K]+ | 9072 | 4 |
| GcGM2 | [M+Na]+ | 9072 | 1 |
| GcGM2 | [M+NH4]+ | 9072 | 0 |
| GcGM3 | [M-H]- | 9072 | 0 |
| GcGM3 | [M+H]+ | 9072 | 0 |
| GcGM3 | [M+K]+ | 9072 | 0 |
| GcGM3 | [M+Na]+ | 9072 | 0 |
| HexCer | [M-H]- | 9072 | 2 |
| HexCer | [M+AcOH-H]- | 9072 | 13 |
| HexCer | [M+Cl]- | 9072 | 6 |
| HexCer | [M+FA-H]- | 9072 | 20 |
| HexCer | [M+H]+ | 9072 | 19 |
| HexCer | [M+K]+ | 9072 | 0 |
| HexCer | [M+Na]+ | 9072 | 4 |
| LacCer | [M-H]- | 9072 | 2 |
| LacCer | [M+AcOH-H]- | 9072 | 0 |
| LacCer | [M+Cl]- | 9072 | 0 |
| LacCer | [M+FA-H]- | 9072 | 0 |
| LacCer | [M+H]+ | 9072 | 5 |
| LacCer | [M+K]+ | 9072 | 0 |
| LacCer | [M+Na]+ | 9072 | 0 |
| LCB | [M+H]+ | 81 | 1 |
| LCB | [M+K]+ | 81 | 0 |
| LCB | [M+Na]+ | 81 | 0 |
| LCB | [M+NH4]+ | 81 | 0 |
| LCB\_P | [M-H]- | 81 | 0 |
| LCB\_P | [M+H]+ | 81 | 0 |
| LPA | [M-H]- | 204 | 1 |
| LPA | [M+AcOH-H]- | 204 | 0 |
| LPA | [M+Cl]- | 204 | 0 |
| LPA | [M+FA-H]- | 204 | 0 |
| LPC | [M-CH3]- | 204 | 5 |
| LPC | [M-H]- | 204 | 1 |
| LPC | [M+AcOH-H]- | 204 | 12 |
| LPC | [M+Cl]- | 204 | 16 |
| LPC | [M+FA-H]- | 204 | 30 |
| LPC | [M+H]+ | 204 | 91 |
| LPC | [M+K]+ | 204 | 17 |
| LPC | [M+Na]+ | 204 | 40 |
| LPC | [M+NH4]+ | 204 | 2 |
| LPE | [M-H]- | 204 | 25 |
| LPE | [M+AcOH-H]- | 204 | 0 |
| LPE | [M+Cl]- | 204 | 0 |
| LPE | [M+FA-H]- | 204 | 0 |
| LPE | [M+H]+ | 204 | 12 |
| LPE | [M+K]+ | 204 | 0 |
| LPE | [M+Na]+ | 204 | 9 |
| LPE | [M+NH4]+ | 204 | 0 |
| LPG | [M-H]- | 204 | 3 |
| LPG | [M+AcOH-H]- | 204 | 0 |
| LPG | [M+Cl]- | 204 | 0 |
| LPG | [M+FA-H]- | 204 | 0 |
| LPG | [M+H]+ | 204 | 0 |
| LPG | [M+K]+ | 204 | 0 |
| LPG | [M+Na]+ | 204 | 0 |
| LPG | [M+NH4]+ | 204 | 0 |
| LPI | [M-H]- | 204 | 12 |
| LPI | [M+AcOH-H]- | 204 | 0 |
| LPI | [M+Cl]- | 204 | 0 |
| LPI | [M+FA-H]- | 204 | 0 |
| LPI | [M+H]+ | 204 | 0 |
| LPI | [M+K]+ | 204 | 0 |
| LPI | [M+Na]+ | 204 | 0 |
| LPI | [M+NH4]+ | 204 | 0 |
| LPS | [M-H]- | 204 | 0 |
| LPS | [M+AcOH-H]- | 204 | 0 |
| LPS | [M+Cl]- | 204 | 0 |
| LPS | [M+FA-H]- | 204 | 0 |
| LPS | [M+H]+ | 204 | 0 |
| LPS | [M+K]+ | 204 | 0 |
| LPS | [M+Na]+ | 204 | 0 |
| LPS | [M+NH4]+ | 204 | 0 |
| LysoCPE | [M-H]- | 81 | 0 |
| LysoCPE | [M+AcOH-H]- | 81 | 0 |
| LysoCPE | [M+Cl]- | 81 | 0 |
| LysoCPE | [M+FA-H]- | 81 | 0 |
| LysoCPI | [M-H]- | 81 | 0 |
| LysoCPI | [M+AcOH-H]- | 81 | 0 |
| LysoCPI | [M+Cl]- | 81 | 0 |
| LysoCPI | [M+FA-H]- | 81 | 0 |
| LysoCPI | [M+H]+ | 81 | 0 |
| LysoCPI | [M+K]+ | 81 | 0 |
| LysoCPI | [M+Na]+ | 81 | 0 |
| LysoCPI | [M+NH4]+ | 81 | 0 |
| LysoHexCer | [M-H]- | 81 | 0 |
| LysoHexCer | [M+AcOH-H]- | 81 | 0 |
| LysoHexCer | [M+Cl]- | 81 | 0 |
| LysoHexCer | [M+FA-H]- | 81 | 0 |
| LysoSM | [M-CH3]- | 81 | 0 |
| LysoSM | [M-H]- | 81 | 0 |
| LysoSM | [M+AcOH-H]- | 81 | 0 |
| LysoSM | [M+Cl]- | 81 | 0 |
| LysoSM | [M+FA-H]- | 81 | 0 |
| LysoSM | [M+H]+ | 81 | 3 |
| LysoSM | [M+K]+ | 81 | 1 |
| LysoSM | [M+Na]+ | 81 | 0 |
| LysoSM | [M+NH4]+ | 81 | 0 |
| MG | [M-H]- | 102 | 0 |
| MG | [M+H]+ | 102 | 0 |
| MG | [M+K]+ | 102 | 0 |
| MG | [M+Na]+ | 102 | 0 |
| MG | [M+NH4]+ | 102 | 0 |
| MGDG | [M+H]+ | 8649 | 0 |
| MGDG | [M+K]+ | 8649 | 0 |
| MGDG | [M+Na]+ | 8649 | 4 |
| MGDG | [M+NH4]+ | 8649 | 0 |
| MIP2C | [M-H]- | 9072 | 0 |
| MIP2C | [M+AcOH-H]- | 9072 | 0 |
| MIP2C | [M+Cl]- | 9072 | 0 |
| MIP2C | [M+FA-H]- | 9072 | 0 |
| MIPC | [M-H]- | 9072 | 0 |
| MIPC | [M+AcOH-H]- | 9072 | 1 |
| MIPC | [M+Cl]- | 9072 | 0 |
| MIPC | [M+FA-H]- | 9072 | 0 |
| MMPE | [M-H]- | 8649 | 0 |
| MMPE | [M+AcOH-H]- | 8649 | 0 |
| MMPE | [M+Cl]- | 8649 | 0 |
| MMPE | [M+FA-H]- | 8649 | 0 |
| MMPE | [M+H]+ | 8649 | 0 |
| MMPE | [M+K]+ | 8649 | 0 |
| MMPE | [M+Na]+ | 8649 | 0 |
| MMPE | [M+NH4]+ | 8649 | 0 |
| PA | [M-H]- | 8649 | 4 |
| PA | [M+AcOH-H]- | 8649 | 1 |
| PA | [M+Cl]- | 8649 | 1 |
| PA | [M+FA-H]- | 8649 | 0 |
| PC | [M-CH3]- | 8649 | 9 |
| PC | [M-H]- | 8649 | 2 |
| PC | [M+AcOH-H]- | 8649 | 48 |
| PC | [M+Cl]- | 8649 | 26 |
| PC | [M+FA-H]- | 8649 | 83 |
| PC | [M+H]+ | 8649 | 166 |
| PC | [M+K]+ | 8649 | 78 |
| PC | [M+Na]+ | 8649 | 116 |
| PC | [M+NH4]+ | 8649 | 18 |
| PE | [M-H]- | 8649 | 43 |
| PE | [M+AcOH-H]- | 8649 | 8 |
| PE | [M+Cl]- | 8649 | 3 |
| PE | [M+FA-H]- | 8649 | 6 |
| PE | [M+H]+ | 8649 | 7 |
| PE | [M+K]+ | 8649 | 4 |
| PE | [M+Na]+ | 8649 | 16 |
| PE | [M+NH4]+ | 8649 | 0 |
| PG | [M-H]- | 8649 | 9 |
| PG | [M+AcOH-H]- | 8649 | 0 |
| PG | [M+Cl]- | 8649 | 0 |
| PG | [M+FA-H]- | 8649 | 0 |
| PG | [M+H]+ | 8649 | 0 |
| PG | [M+K]+ | 8649 | 0 |
| PG | [M+Na]+ | 8649 | 1 |
| PG | [M+NH4]+ | 8649 | 0 |
| PI | [M-H]- | 8649 | 47 |
| PI | [M+AcOH-H]- | 8649 | 8 |
| PI | [M+Cl]- | 8649 | 4 |
| PI | [M+FA-H]- | 8649 | 2 |
| PI | [M+H]+ | 8649 | 0 |
| PI | [M+K]+ | 8649 | 7 |
| PI | [M+Na]+ | 8649 | 18 |
| PI | [M+NH4]+ | 8649 | 1 |
| PS | [M-H]- | 8649 | 2 |
| PS | [M+AcOH-H]- | 8649 | 1 |
| PS | [M+Cl]- | 8649 | 0 |
| PS | [M+FA-H]- | 8649 | 0 |
| PS | [M+H]+ | 8649 | 0 |
| PS | [M+K]+ | 8649 | 0 |
| PS | [M+Na]+ | 8649 | 1 |
| PS | [M+NH4]+ | 8649 | 2 |
| SM | [M-CH3]- | 9072 | 0 |
| SM | [M-H]- | 9072 | 0 |
| SM | [M+AcOH-H]- | 9072 | 11 |
| SM | [M+Cl]- | 9072 | 2 |
| SM | [M+FA-H]- | 9072 | 16 |
| SM | [M+H]+ | 9072 | 215 |
| SM | [M+K]+ | 9072 | 40 |
| SM | [M+Na]+ | 9072 | 102 |
| SM | [M+NH4]+ | 9072 | 10 |
| Sulfatide | [M-H]- | 9072 | 0 |
| Sulfatide | [M+AcOH-H]- | 9072 | 0 |
| Sulfatide | [M+Cl]- | 9072 | 0 |
| Sulfatide | [M+FA-H]- | 9072 | 0 |
| Taurine | [M-H]- | 204 | 0 |
| Taurine | [M+AcOH-H]- | 204 | 0 |
| Taurine | [M+Cl]- | 204 | 0 |
| Taurine | [M+FA-H]- | 204 | 0 |
| Taurine | [M+H]+ | 204 | 0 |
| Taurine | [M+K]+ | 204 | 0 |
| Taurine | [M+Na]+ | 204 | 0 |
| Taurine | [M+NH4]+ | 204 | 0 |
| TG | [M+K]+ | 59319 | 1 |
| TG | [M+Li]+ | 59319 | 4 |
| TG | [M+Na]+ | 59319 | 78 |
| TG | [M+NH4]+ | 59319 | 143 |

## MS-DIAL Library Contents

```
knitr::kable(msdial_all_counts, "html") %>%
  kableExtra::kable_styling(bootstrap_options = c("striped", "hover", "responsive"), position = "left", full_width = FALSE) %>%
  kableExtra::row_spec(1:nrow(msdial_all_counts), bold = T, color = "#CCCCCC", background = "#990066",font_size = "xx-large")
```

| msdial\_lipidClass\_adj | msdial\_adduct | msdial\_count | msdial\_id\_count |
| --- | --- | --- | --- |
| ACar | [M]+ | 102 | 20 |
| AcGM3 | [M-H]- | 184 | 3 |
| AcGM3 | [M+NH4]+ | 2346 | 0 |
| AcylGlcADG | [M-H]- | 10206 | 0 |
| AcylGlcADG | [M+NH4]+ | 10206 | 1 |
| Alkyl\_PC | [M+AcOH-H]- | 1098 | 26 |
| Alkyl\_PC | [M+FA-H]- | 1098 | 29 |
| Alkyl\_PC | [M+H]+ | 1098 | 66 |
| Alkyl\_PE | [M-H]- | 1098 | 39 |
| Alkyl\_PE | [M+H]+ | 1098 | 28 |
| BMP | [M+NH4]+ | 1891 | 5 |
| CE | [M+NH4]+ | 28 | 20 |
| Cer\_ADS | [M-H]- | 1173 | 0 |
| Cer\_ADS | [M+AcOH-H]- | 1173 | 1 |
| Cer\_ADS | [M+FA-H]- | 1173 | 0 |
| Cer\_AP | [M-H]- | 3519 | 1 |
| Cer\_AP | [M+AcOH-H]- | 3519 | 1 |
| Cer\_AP | [M+FA-H]- | 3519 | 0 |
| Cer\_BDS | [M-H]- | 1173 | 0 |
| Cer\_BDS | [M+AcOH-H]- | 1173 | 1 |
| Cer\_BDS | [M+FA-H]- | 1173 | 0 |
| Cer\_BS | [M-H]- | 3519 | 1 |
| Cer\_BS | [M+AcOH-H]- | 3519 | 3 |
| Cer\_BS | [M+FA-H]- | 3519 | 0 |
| Cer\_EODS | [M-H]- | 29325 | 0 |
| Cer\_EODS | [M+AcOH-H]- | 29325 | 0 |
| Cer\_EODS | [M+FA-H]- | 29325 | 0 |
| Cer\_EOS | [M-H]- | 29325 | 0 |
| Cer\_EOS | [M+AcOH-H]- | 29325 | 0 |
| Cer\_EOS | [M+FA-H]- | 29325 | 1 |
| Ceramide | [M-H]- | 11730 | 13 |
| Ceramide | [M+AcOH-H]- | 11730 | 53 |
| Ceramide | [M+FA-H]- | 11730 | 26 |
| Ceramide | [M+H]+ | 4692 | 36 |
| CL | [M-H]- | 14252 | 4 |
| CL | [M+NH4]+ | 14252 | 0 |
| DG | [M+NH4]+ | 1891 | 12 |
| DGDG | [M+AcOH-H]- | 1891 | 0 |
| DGDG | [M+FA-H]- | 1891 | 0 |
| DGDG | [M+NH4]+ | 1891 | 0 |
| DGTS | [M+H]+ | 1891 | 0 |
| EtherOxPC | [M+AcOH-H]- | 105 | 0 |
| EtherOxPC | [M+FA-H]- | 105 | 0 |
| EtherOxPE | [M-H]- | 105 | 0 |
| FA | [M-H]- | 43 | 0 |
| FAHFA | [M-H]- | 3299 | 18 |
| GlcADG | [M-H]- | 1326 | 4 |
| GlcADG | [M+NH4]+ | 1326 | 1 |
| HBMP | [M-H]- | 10206 | 0 |
| HBMP | [M+NH4]+ | 10206 | 5 |
| HexCer | [M-H]- | 8211 | 0 |
| HexCer | [M+AcOH-H]- | 8211 | 11 |
| HexCer | [M+FA-H]- | 8211 | 16 |
| HexCer | [M+H]+ | 4692 | 20 |
| LDGTS | [M+H]+ | 60 | 0 |
| LPA | [M-H]- | 61 | 1 |
| LPC | [M+AcOH-H]- | 61 | 22 |
| LPC | [M+FA-H]- | 61 | 29 |
| LPC | [M+H]+ | 61 | 70 |
| LPE | [M-H]- | 61 | 26 |
| LPE | [M+H]+ | 61 | 3 |
| LPG | [M-H]- | 61 | 3 |
| LPI | [M-H]- | 61 | 14 |
| LPS | [M-H]- | 61 | 1 |
| MAG | [M+NH4]+ | 61 | 0 |
| MGDG | [M+AcOH-H]- | 1891 | 1 |
| MGDG | [M+FA-H]- | 1891 | 2 |
| MGDG | [M+NH4]+ | 1891 | 1 |
| OxPC | [M+AcOH-H]- | 105 | 3 |
| OxPC | [M+FA-H]- | 105 | 4 |
| OxPE | [M-H]- | 105 | 2 |
| OxPG | [M-H]- | 105 | 2 |
| OxPI | [M-H]- | 105 | 2 |
| OxPS | [M-H]- | 105 | 0 |
| PA | [M-H]- | 1891 | 11 |
| PC | [M+AcOH-H]- | 1891 | 60 |
| PC | [M+FA-H]- | 1891 | 88 |
| PC | [M+H]+ | 1891 | 188 |
| PE | [M-H]- | 1891 | 57 |
| PE | [M+H]+ | 1891 | 20 |
| PEtOH | [M-H]- | 1326 | 0 |
| PEtOH | [M+NH4]+ | 1326 | 3 |
| PG | [M-H]- | 1891 | 9 |
| PG | [M+NH4]+ | 1891 | 0 |
| PI | [M-H]- | 1891 | 48 |
| PMeOH | [M-H]- | 1326 | 0 |
| PMeOH | [M+NH4]+ | 1326 | 0 |
| PS | [M-H]- | 1891 | 5 |
| PS | [M+H]+ | 1891 | 1 |
| SHexCer | [M-H]- | 184 | 1 |
| SHexCer | [M+H]+ | 2346 | 0 |
| SM | [M+AcOH-H]- | 4692 | 22 |
| SM | [M+FA-H]- | 4692 | 35 |
| SM | [M+H]+ | 4692 | 60 |
| SQDG | [M-H]- | 1326 | 1 |
| SQDG | [M+NH4]+ | 1326 | 0 |
| TG | [M+Na]+ | 32509 | 119 |
| TG | [M+NH4]+ | 32509 | 224 |

## Both Library Contents

Class + Adduct Present in both Calico and MS-DIAL lipid libraries.

```
knitr::kable(both_libs, "html") %>%
  kableExtra::kable_styling(bootstrap_options = c("striped", "hover", "responsive"), position = "left", full_width = FALSE) %>%
  kableExtra::row_spec(1:nrow(both_libs), bold = T, color = "#CCCCCC", background = "#990066",font_size = "xx-large")
```

| Lipid Class | Adduct | calico\_count | calico\_id\_count | msdial\_count | msdial\_id\_count |
| --- | --- | --- | --- | --- | --- |
| AcGM3 | [M-H]- | 9072 | 1 | 184 | 3 |
| Alkyl\_PC | [M+AcOH-H]- | 3627 | 17 | 1098 | 26 |
| Alkyl\_PC | [M+FA-H]- | 3627 | 20 | 1098 | 29 |
| Alkyl\_PC | [M+H]+ | 3627 | 81 | 1098 | 66 |
| Alkyl\_PE | [M-H]- | 3627 | 53 | 1098 | 39 |
| Alkyl\_PE | [M+H]+ | 3627 | 31 | 1098 | 28 |
| CE | [M+NH4]+ | 204 | 0 | 28 | 20 |
| Ceramide | [M-H]- | 9072 | 6 | 11730 | 13 |
| Ceramide | [M+AcOH-H]- | 9072 | 31 | 11730 | 53 |
| Ceramide | [M+FA-H]- | 9072 | 19 | 11730 | 26 |
| Ceramide | [M+H]+ | 9072 | 34 | 4692 | 36 |
| DG | [M+NH4]+ | 8649 | 0 | 1891 | 12 |
| DGDG | [M+AcOH-H]- | 8649 | 0 | 1891 | 0 |
| DGDG | [M+FA-H]- | 8649 | 0 | 1891 | 0 |
| DGDG | [M+NH4]+ | 8649 | 0 | 1891 | 0 |
| DGTS | [M+H]+ | 8649 | 0 | 1891 | 0 |
| FA | [M-H]- | 204 | 0 | 43 | 0 |
| FAHFA | [M-H]- | 8649 | 2 | 3299 | 18 |
| HexCer | [M-H]- | 9072 | 2 | 8211 | 0 |
| HexCer | [M+AcOH-H]- | 9072 | 13 | 8211 | 11 |
| HexCer | [M+FA-H]- | 9072 | 20 | 8211 | 16 |
| HexCer | [M+H]+ | 9072 | 19 | 4692 | 20 |
| LPA | [M-H]- | 204 | 1 | 61 | 1 |
| LPC | [M+AcOH-H]- | 204 | 12 | 61 | 22 |
| LPC | [M+FA-H]- | 204 | 30 | 61 | 29 |
| LPC | [M+H]+ | 204 | 91 | 61 | 70 |
| LPE | [M-H]- | 204 | 25 | 61 | 26 |
| LPE | [M+H]+ | 204 | 12 | 61 | 3 |
| LPG | [M-H]- | 204 | 3 | 61 | 3 |
| LPI | [M-H]- | 204 | 12 | 61 | 14 |
| LPS | [M-H]- | 204 | 0 | 61 | 1 |
| MGDG | [M+NH4]+ | 8649 | 0 | 1891 | 1 |
| PA | [M-H]- | 8649 | 4 | 1891 | 11 |
| PC | [M+AcOH-H]- | 8649 | 48 | 1891 | 60 |
| PC | [M+FA-H]- | 8649 | 83 | 1891 | 88 |
| PC | [M+H]+ | 8649 | 166 | 1891 | 188 |
| PE | [M-H]- | 8649 | 43 | 1891 | 57 |
| PE | [M+H]+ | 8649 | 7 | 1891 | 20 |
| PG | [M-H]- | 8649 | 9 | 1891 | 9 |
| PG | [M+NH4]+ | 8649 | 0 | 1891 | 0 |
| PI | [M-H]- | 8649 | 47 | 1891 | 48 |
| PS | [M-H]- | 8649 | 2 | 1891 | 5 |
| PS | [M+H]+ | 8649 | 0 | 1891 | 1 |
| SM | [M+AcOH-H]- | 9072 | 11 | 4692 | 22 |
| SM | [M+FA-H]- | 9072 | 16 | 4692 | 35 |
| SM | [M+H]+ | 9072 | 215 | 4692 | 60 |
| TG | [M+Na]+ | 59319 | 78 | 32509 | 119 |
| TG | [M+NH4]+ | 59319 | 143 | 32509 | 224 |

## Both Library Contents and Identified

Class + Adduct Present in both Calico and MS-DIAL lipid libraries, identified at least once.

```
knitr::kable(both_libs_identified, "html") %>%
  kableExtra::kable_styling(bootstrap_options = c("striped", "hover", "responsive"), position = "left", full_width = FALSE) %>%
  kableExtra::row_spec(1:nrow(both_libs_identified), bold = T, color = "#CCCCCC", background = "#990066",font_size = "xx-large")
```

| Lipid Class | Adduct | calico\_count | calico\_id\_count | msdial\_count | msdial\_id\_count |
| --- | --- | --- | --- | --- | --- |
| AcGM3 | [M-H]- | 9072 | 1 | 184 | 3 |
| Alkyl\_PC | [M+AcOH-H]- | 3627 | 17 | 1098 | 26 |
| Alkyl\_PC | [M+FA-H]- | 3627 | 20 | 1098 | 29 |
| Alkyl\_PC | [M+H]+ | 3627 | 81 | 1098 | 66 |
| Alkyl\_PE | [M-H]- | 3627 | 53 | 1098 | 39 |
| Alkyl\_PE | [M+H]+ | 3627 | 31 | 1098 | 28 |
| CE | [M+NH4]+ | 204 | 0 | 28 | 20 |
| Ceramide | [M-H]- | 9072 | 6 | 11730 | 13 |
| Ceramide | [M+AcOH-H]- | 9072 | 31 | 11730 | 53 |
| Ceramide | [M+FA-H]- | 9072 | 19 | 11730 | 26 |
| Ceramide | [M+H]+ | 9072 | 34 | 4692 | 36 |
| DG | [M+NH4]+ | 8649 | 0 | 1891 | 12 |
| FAHFA | [M-H]- | 8649 | 2 | 3299 | 18 |
| HexCer | [M-H]- | 9072 | 2 | 8211 | 0 |
| HexCer | [M+AcOH-H]- | 9072 | 13 | 8211 | 11 |
| HexCer | [M+FA-H]- | 9072 | 20 | 8211 | 16 |
| HexCer | [M+H]+ | 9072 | 19 | 4692 | 20 |
| LPA | [M-H]- | 204 | 1 | 61 | 1 |
| LPC | [M+AcOH-H]- | 204 | 12 | 61 | 22 |
| LPC | [M+FA-H]- | 204 | 30 | 61 | 29 |
| LPC | [M+H]+ | 204 | 91 | 61 | 70 |
| LPE | [M-H]- | 204 | 25 | 61 | 26 |
| LPE | [M+H]+ | 204 | 12 | 61 | 3 |
| LPG | [M-H]- | 204 | 3 | 61 | 3 |
| LPI | [M-H]- | 204 | 12 | 61 | 14 |
| LPS | [M-H]- | 204 | 0 | 61 | 1 |
| MGDG | [M+NH4]+ | 8649 | 0 | 1891 | 1 |
| PA | [M-H]- | 8649 | 4 | 1891 | 11 |
| PC | [M+AcOH-H]- | 8649 | 48 | 1891 | 60 |
| PC | [M+FA-H]- | 8649 | 83 | 1891 | 88 |
| PC | [M+H]+ | 8649 | 166 | 1891 | 188 |
| PE | [M-H]- | 8649 | 43 | 1891 | 57 |
| PE | [M+H]+ | 8649 | 7 | 1891 | 20 |
| PG | [M-H]- | 8649 | 9 | 1891 | 9 |
| PI | [M-H]- | 8649 | 47 | 1891 | 48 |
| PS | [M-H]- | 8649 | 2 | 1891 | 5 |
| PS | [M+H]+ | 8649 | 0 | 1891 | 1 |
| SM | [M+AcOH-H]- | 9072 | 11 | 4692 | 22 |
| SM | [M+FA-H]- | 9072 | 16 | 4692 | 35 |
| SM | [M+H]+ | 9072 | 215 | 4692 | 60 |
| TG | [M+Na]+ | 59319 | 78 | 32509 | 119 |
| TG | [M+NH4]+ | 59319 | 143 | 32509 | 224 |

## Calico Only Library Counts

Identified class + adduct from Calico libraries, class + adduct is not present in MS-DIAL libraries.

```
knitr::kable(calico_only_libs, "html") %>%
  kableExtra::kable_styling(bootstrap_options = c("striped", "hover", "responsive"), position = "left", full_width = FALSE) %>%
  kableExtra::row_spec(1:nrow(calico_only_libs), bold = T, color = "#CCCCCC", background = "#990066",font_size = "xx-large")
```

| Lipid Class | Adduct | calico\_count | calico\_id\_count |
| --- | --- | --- | --- |
| AcGM3 | [M+H]+ | 9072 | 2 |
| Alkyl\_PC | [M+Cl]- | 3627 | 1 |
| Alkyl\_PC | [M+K]+ | 3627 | 1 |
| Alkyl\_PC | [M+Na]+ | 3627 | 20 |
| Alkyl\_PE | [M+AcOH-H]- | 3627 | 1 |
| Alkyl\_PE | [M+K]+ | 3627 | 6 |
| Alkyl\_PE | [M+Na]+ | 3627 | 23 |
| APCS | [M+H]+ | 204 | 3 |
| BMP | [M-H]- | 8649 | 1 |
| Carn | [M+H]+ | 204 | 23 |
| CDP\_DG | [M-H]- | 8649 | 1 |
| CE | [M+Na]+ | 204 | 4 |
| CPE | [M+H]+ | 9072 | 2 |
| CPE | [M+Na]+ | 9072 | 7 |
| CPI | [M-H]- | 9072 | 3 |
| CPI | [M+AcOH-H]- | 9072 | 4 |
| CPI | [M+Cl]- | 9072 | 2 |
| DG | [M+Na]+ | 8649 | 10 |
| DMPE | [M+H]+ | 8649 | 1 |
| GB3 | [M+H]+ | 9072 | 2 |
| GcGM2 | [M-H]- | 9072 | 7 |
| GcGM2 | [M+H]+ | 9072 | 6 |
| GcGM2 | [M+K]+ | 9072 | 4 |
| GcGM2 | [M+Na]+ | 9072 | 1 |
| HexCer | [M+Cl]- | 9072 | 6 |
| HexCer | [M+Na]+ | 9072 | 4 |
| LacCer | [M-H]- | 9072 | 2 |
| LacCer | [M+H]+ | 9072 | 5 |
| LCB | [M+H]+ | 81 | 1 |
| LPC | [M-CH3]- | 204 | 5 |
| LPC | [M-H]- | 204 | 1 |
| LPC | [M+Cl]- | 204 | 16 |
| LPC | [M+K]+ | 204 | 17 |
| LPC | [M+Na]+ | 204 | 40 |
| LPC | [M+NH4]+ | 204 | 2 |
| LPE | [M+Na]+ | 204 | 9 |
| LysoSM | [M+H]+ | 81 | 3 |
| LysoSM | [M+K]+ | 81 | 1 |
| MGDG | [M+Na]+ | 8649 | 4 |
| MIPC | [M+AcOH-H]- | 9072 | 1 |
| PA | [M+AcOH-H]- | 8649 | 1 |
| PA | [M+Cl]- | 8649 | 1 |
| PC | [M-CH3]- | 8649 | 9 |
| PC | [M-H]- | 8649 | 2 |
| PC | [M+Cl]- | 8649 | 26 |
| PC | [M+K]+ | 8649 | 78 |
| PC | [M+Na]+ | 8649 | 116 |
| PC | [M+NH4]+ | 8649 | 18 |
| PE | [M+AcOH-H]- | 8649 | 8 |
| PE | [M+Cl]- | 8649 | 3 |
| PE | [M+FA-H]- | 8649 | 6 |
| PE | [M+K]+ | 8649 | 4 |
| PE | [M+Na]+ | 8649 | 16 |
| PG | [M+Na]+ | 8649 | 1 |
| PI | [M+AcOH-H]- | 8649 | 8 |
| PI | [M+Cl]- | 8649 | 4 |
| PI | [M+FA-H]- | 8649 | 2 |
| PI | [M+K]+ | 8649 | 7 |
| PI | [M+Na]+ | 8649 | 18 |
| PI | [M+NH4]+ | 8649 | 1 |
| PS | [M+AcOH-H]- | 8649 | 1 |
| PS | [M+Na]+ | 8649 | 1 |
| PS | [M+NH4]+ | 8649 | 2 |
| SM | [M+Cl]- | 9072 | 2 |
| SM | [M+K]+ | 9072 | 40 |
| SM | [M+Na]+ | 9072 | 102 |
| SM | [M+NH4]+ | 9072 | 10 |
| TG | [M+K]+ | 59319 | 1 |
| TG | [M+Li]+ | 59319 | 4 |

## MS-DIAL Only Library Counts

Identified class + adduct from MS-DIAL libraries, class + adduct is not present in Calico libraries.

```
knitr::kable(msdial_only_libs, "html") %>%
  kableExtra::kable_styling(bootstrap_options = c("striped", "hover", "responsive"), position = "left", full_width = FALSE) %>%
  kableExtra::row_spec(1:nrow(msdial_only_libs), bold = T, color = "#CCCCCC", background = "#990066",font_size = "xx-large")
```

| Lipid Class | Adduct | msdial\_count | msdial\_id\_count |
| --- | --- | --- | --- |
| ACar | [M]+ | 102 | 20 |
| AcylGlcADG | [M+NH4]+ | 10206 | 1 |
| BMP | [M+NH4]+ | 1891 | 5 |
| Cer\_ADS | [M+AcOH-H]- | 1173 | 1 |
| Cer\_AP | [M-H]- | 3519 | 1 |
| Cer\_AP | [M+AcOH-H]- | 3519 | 1 |
| Cer\_BDS | [M+AcOH-H]- | 1173 | 1 |
| Cer\_BS | [M-H]- | 3519 | 1 |
| Cer\_BS | [M+AcOH-H]- | 3519 | 3 |
| Cer\_EOS | [M+FA-H]- | 29325 | 1 |
| CL | [M-H]- | 14252 | 4 |
| GlcADG | [M-H]- | 1326 | 4 |
| GlcADG | [M+NH4]+ | 1326 | 1 |
| HBMP | [M+NH4]+ | 10206 | 5 |
| MGDG | [M+AcOH-H]- | 1891 | 1 |
| MGDG | [M+FA-H]- | 1891 | 2 |
| OxPC | [M+AcOH-H]- | 105 | 3 |
| OxPC | [M+FA-H]- | 105 | 4 |
| OxPE | [M-H]- | 105 | 2 |
| OxPG | [M-H]- | 105 | 2 |
| OxPI | [M-H]- | 105 | 2 |
| PEtOH | [M+NH4]+ | 1326 | 3 |
| SHexCer | [M-H]- | 184 | 1 |
| SQDG | [M-H]- | 1326 | 1 |

```
knitr::knit_exit()
```
