# Supplementary material for: MAVEN2: An Updated Open-Source Mass Spectrometry Exploration Platform
Source: Metabolites. 2022 Jul 25;12(8):684. doi: 10.3390/metabo12080684 (PMC9330773; doi:10.3390/metabo12080684)
Supplement: Supplementary file 1 [file metabolites-12-00684-s001.zip › metabolites-1827771_proof-supplementary/Dataset_S4_Metabolomics_Analysis_Script.zip!/S12_Metabolomics_Analysis_Script/S12_Metabolomics_Analysis_Script.html]

MAVEN Paper Metabolites Analysis


# MAVEN Paper Metabolites Analysis

#### Eugene Melamud, Phillip Seitzer

# Search information

# Peak Detection Parameters

# Peak Scoring Parameters

# Analysis Functions

Function to retrieve experiment results, categorize appropriately

## Import Experimental Results f(x)

Experimental information is spread across three tables: samples, peaks, and peakgroups. Information is linked across these three tables via sampleId, peakId, and groupId columns. Compound annotations are annotated as correct or incorrect either by quant value (water samples), or RT (yeast samples). Once a compound has been found to be correct in a water sample, the RT is noted, and used to assess correctness in the yeast samples.

```
get_experiment_data_table <- function(mzroll_db_file) {
  con <- DBI::dbConnect(RSQLite::SQLite(), dbname = mzroll_db_file)
  
  samples <- tbl(con, "samples") %>%
    collect() %>%
    dplyr::mutate(components = stringr::str_split(name,"[_\\.]")) %>%
    dplyr::mutate(type = sapply(components, function(x){x[6]})) %>%
    dplyr::mutate(sample_num = sapply(components, function(x){as.numeric(x[7])})) %>%
    dplyr::select(sampleId, name, type, sample_num)
  
  peakgroups <- tbl(con, "peakgroups") %>%
    collect() %>%
    dplyr::mutate(compoundName = toupper(compoundName)) %>%
    dplyr::select(groupId, compoundName, adductName, ms2Score)
  
  peaks=tbl(con, "peaks") %>%
    collect() %>%
    dplyr::select(peakId, groupId, sampleId, peakAreaTop, rt)
    
  DBI::dbDisconnect(con)
  
  # organize data from mzrolldb file to prepare for search  
  data = peaks %>%
    left_join(peakgroups, by="groupId") %>% 
    left_join(samples, by ="sampleId") %>%
    dplyr::group_by(groupId) %>%
    dplyr::mutate(group_rt = median(rt)) %>%
    dplyr::ungroup() %>%
    dplyr::rename(peak_rt = rt) %>%
    dplyr::select(groupId, compoundName, adductName, ms2Score, group_rt, type, sample_num, peakAreaTop, 
                  peak_rt)
}

get_experiment_results <- function(mzroll_db_file,
                                   standards,
                                   rt_table=NULL,
                                   analysis_function) {
   # organize data from mzrolldb file to prepare for search
  data = get_experiment_data_table(mzroll_db_file = mzroll_db_file)
  
   # run search
  results_table <- do.call(analysis_function, args=list(data, standards, rt_table))
}
```

Top Match: how often is the top-scoring MS2 peakgroup for a standard compound correct? If all peakgroups have an MS2 score below the threshold, they Standard compounds that were not identified are not considered. Compounds that were not spiked in standards were not considered.

## Quant top match f(x)

```
correct_top_match_by_quant <- function(data, standards, rt_table=NULL, quant_ratio_threshold=2) {
  
  matched_data <- dplyr::left_join(data, standards, by = c("compoundName")) %>%
    dplyr::mutate(is_standard = !is.na(sample_num_1) & !is.na(sample_num_2))
  
  matched_standards <- matched_data %>%
    dplyr::filter(is_standard==TRUE) %>%
    dplyr::select(-is_standard)
  
  matched_standards_nested <- matched_standards %>%
    dplyr::group_by(groupId, compoundName, ms2Score, group_rt, sample_num_1, sample_num_2) %>%
    tidyr::nest()
  
  # default to false
  quant_results <- tibble::tibble(groupId = matched_standards_nested$groupId,
                                  median_std_peakAreaTop = rep(NA, length(matched_standards_nested$groupId)),
                                  is_quant_agreement = rep(FALSE, length(matched_standards_nested$groupId)))
  
  # median value in spike-in samples must be at quant_ratio_threshold this amt greater than median value in non-spike in samples
  
  # check for quant agreement
  for(i in 1:nrow(matched_standards_nested)) {
    
    sample_num_1 <- matched_standards_nested$sample_num_1[i]
    sample_num_2 <- matched_standards_nested$sample_num_2[i]
    
    # quant values for expected standard samples
    quant_data_std <- matched_standards_nested$data[[i]] %>%
      dplyr::filter(sample_num %in% c(sample_num_1, sample_num_2) & type == "water")
    
    # quant values for all other samples
    quant_data_other <- matched_standards_nested$data[[i]] %>%
      dplyr::filter(!sample_num %in% c(sample_num_1, sample_num_2) | type != "water")
    
    # must be detected in both spike-in samples
    if (nrow(quant_data_std) == 2) {
      std_quant <- median(quant_data_std$peakAreaTop) 
      
      # choose LOD value here for baseline
      other_quant <- 4096 
      
      # If only detected in spike-in samples, agreement
      if(nrow(quant_data_other) == 0) {
        quant_results$is_quant_agreement[i] <- TRUE
        
      # May be detected in non-spike-in samples (e.g., carryover), but must be much lower concentration 
      } else {
        other_quant <- max(other_quant, median(quant_data_other$peakAreaTop))
      }
      
      quant_results$median_std_peakAreaTop[i] <- std_quant
      quant_results$is_quant_agreement[i] <- (std_quant/other_quant) > quant_ratio_threshold
    }
    
  }
  
  # Determine if the max ms2 agrees with the quant profile
  compound_recovery <- dplyr::inner_join(matched_standards, quant_results, by = c("groupId")) %>%
    dplyr::group_by(compoundName, adductName) %>%
    tidyr::nest()
  
  N <- nrow(compound_recovery)
  output <- tibble::tibble(compoundName = compound_recovery$compoundName,
                           adductName = compound_recovery$adductName,
                           compound_rt = rep(NA, N),
                           ms2Score = rep(NA, N),
                           is_correct = rep(FALSE, N))
  
    for (i in 1:N) {
      
      compound_data <- compound_recovery$data[[i]] %>%
        dplyr::select(ms2Score, group_rt, is_quant_agreement) %>%
        unique()
      
      max_ms2 <- max(compound_data$ms2Score)
      
      output$ms2Score[i] <- max_ms2
      
      for (j in 1:nrow(compound_data)) {
        
        if (compound_data$is_quant_agreement[j] == TRUE) {
          
            output$compound_rt[i] <- compound_data$group_rt[j]
            
            if (compound_data$ms2Score[j] == max_ms2) {
              output$is_correct[i] <- TRUE
              break
            }
        }
      }
      
    }
  
  output_filtered <- output %>%
    dplyr::filter(!is.na(compound_rt))
}
```

## Quant enrichment f(x)

```
correct_enrichment_by_quant <- function(data, standards, rt_table=NULL, quant_ratio_threshold=2) {
  matched_data <- dplyr::left_join(data, standards, by = c("compoundName")) %>%
    dplyr::mutate(is_standard = !is.na(sample_num_1) & !is.na(sample_num_2))
  
  matched_standards <- matched_data %>%
    dplyr::filter(is_standard==TRUE) %>%
    dplyr::select(-is_standard)
  
  matched_standards_nested <- matched_standards %>%
    dplyr::group_by(groupId, compoundName, ms2Score, group_rt, sample_num_1, sample_num_2) %>%
    tidyr::nest()
  
  # default to false
  quant_results <- tibble::tibble(groupId = matched_standards_nested$groupId,
                                  median_std_peakAreaTop = rep(NA, length(matched_standards_nested$groupId)),
                                  is_quant_agreement = rep(FALSE, length(matched_standards_nested$groupId)))
  
  # median value in spike-in samples must be at quant_ratio_threshold this amt greater than median value in non-spike in samples
  
  # check for quant agreement
  for(i in 1:nrow(matched_standards_nested)) {
    
    sample_num_1 <- matched_standards_nested$sample_num_1[i]
    sample_num_2 <- matched_standards_nested$sample_num_2[i]
    
    # quant values for expected standard samples
    quant_data_std <- matched_standards_nested$data[[i]] %>%
      dplyr::filter(sample_num %in% c(sample_num_1, sample_num_2) & type == "water")
    
    # quant values for all other samples
    quant_data_other <- matched_standards_nested$data[[i]] %>%
      dplyr::filter(!sample_num %in% c(sample_num_1, sample_num_2) | type != "water")
    
    # must be detected in both spike-in samples
    if (nrow(quant_data_std) == 2) {
      std_quant <- median(quant_data_std$peakAreaTop) 
      
      # choose LOD value here for baseline
      other_quant <- 4096 
      
      # If only detected in spike-in samples, agreement
      if(nrow(quant_data_other) == 0) {
        quant_results$is_quant_agreement[i] <- TRUE
        
      # May be detected in non-spike-in samples (e.g., carryover), but must be much lower concentration 
      } else {
        other_quant <- max(other_quant, median(quant_data_other$peakAreaTop))
      }
      
      quant_results$median_std_peakAreaTop[i] <- std_quant
      quant_results$is_quant_agreement[i] <- (std_quant/other_quant) > quant_ratio_threshold
    }
    
  }
  
  # Only consider standards that were actually found
  valid_standards <- dplyr::inner_join(matched_standards, quant_results, by = c("groupId")) %>%
    dplyr::filter(is_quant_agreement == TRUE) %>%
    dplyr::select(compoundName, adductName) %>%
    unique()
  
  # Determine if the max ms2 agrees with the quant profile
  enrichment_results <- dplyr::inner_join(matched_standards, quant_results, by = c("groupId")) %>%
    dplyr::inner_join(valid_standards, by = c("compoundName", "adductName")) %>%
    dplyr::rename(is_correct = is_quant_agreement) %>%
    dplyr::select(groupId, ms2Score, is_correct) %>%
    unique()
}
```

## RT enrichment f(x)

```
correct_enrichment_by_rt <- function(data, standards, rt_table, rt_diff_threshold=0.5) {
  
  matched_data <- dplyr::left_join(data, standards, by = c("compoundName")) %>%
    dplyr::mutate(is_standard = !is.na(sample_num_1) & !is.na(sample_num_2))
   
  matched_standards <- matched_data %>%
    dplyr::filter(is_standard==TRUE) %>%
    dplyr::select(-is_standard) %>%
    dplyr::inner_join(rt_table, by = c("compoundName","adductName")) %>%
    dplyr::mutate(rt_diff = abs(compound_rt-group_rt)) %>%
    dplyr::mutate(is_rt_match = rt_diff <= rt_diff_threshold)
    
  data_results <- matched_standards %>%
    dplyr::rename(is_correct = is_rt_match) %>%
    dplyr::select(groupId, ms2Score, is_correct) %>%
    unique()
  
}
```

## RT top match f(x)

```
correct_top_match_by_rt <- function(data, standards, rt_table, rt_diff_threshold=0.5) {
  
  matched_data <- dplyr::left_join(data, standards, by = c("compoundName")) %>%
    dplyr::mutate(is_standard = !is.na(sample_num_1) & !is.na(sample_num_2))
   
  matched_standards <- matched_data %>%
    dplyr::filter(is_standard==TRUE) %>%
    dplyr::select(-is_standard) %>%
    dplyr::select(compoundName, adductName, ms2Score, group_rt) %>%
    unique() %>%
    dplyr::inner_join(rt_table, by = c("compoundName","adductName")) %>%
    dplyr::mutate(rt_diff = abs(compound_rt-group_rt)) %>%
    dplyr::mutate(is_rt_match = rt_diff <= rt_diff_threshold) %>%
    dplyr::select(compoundName, adductName, ms2Score, is_rt_match)
  
  output <- matched_standards %>%
    dplyr::group_by(compoundName, adductName) %>%
    dplyr::mutate(max_ms2Score = max(ms2Score)) %>%
    dplyr::ungroup() %>%
    dplyr::mutate(is_correct_pg = is_rt_match & ms2Score == max_ms2Score) %>%
    dplyr::group_by(compoundName, adductName, max_ms2Score) %>%
    dplyr::mutate(is_correct = any(is_correct_pg)) %>%
    dplyr::ungroup() %>%
    dplyr::select(-ms2Score) %>%
    dplyr::rename(ms2Score = max_ms2Score) %>%
    dplyr::select(compoundName, adductName, ms2Score, is_correct) %>%
    unique()
    
}
```

## Combine modes f(x)

```
combine_modes <-function(neg_results, pos_results) {
  neg_results_w_mode <- neg_results %>% dplyr::mutate(mode = "neg")
  pos_results_w_mode <- pos_results %>% dplyr::mutate(mode = "pos")
  
  rbind(neg_results_w_mode, pos_results_w_mode)
}
```

## RT Table from water results f(x)

```
get_rt_table <- function(water_results) {
  rt_table <- water_results %>%
    dplyr::select(compoundName, adductName, compound_rt, mode) %>%
    dplyr::arrange(compoundName, adductName, mode)
}
```

## TPR Results f(x)

```
get_tpr_results <- function(results, thresholds){
  
  N <- length(thresholds)
  tpr_results <- tibble::tibble(threshold=thresholds,
                                TP = rep(0, N),
                                FP = rep(0, N),
                                TPR = rep(0, N))
  
  for (i in 1:length(thresholds)){
    threshold <- thresholds[i]
    
    results_tbl <- results %>% dplyr::filter(ms2Score >= threshold)
    
    num_TP <- results_tbl %>% dplyr::filter(is_correct == TRUE) %>% nrow()
    num_FP <- results_tbl %>% dplyr::filter(is_correct == FALSE) %>% nrow()
    
    tpr <- num_TP / (num_TP + num_FP)
    
    tpr_results$TP[i] <- num_TP
    tpr_results$FP[i] <- num_FP
    tpr_results$TPR[i] <- tpr
    
  }
  
  tpr_results
}
```

Import standards data and libraries, correcting for any small naming differences between compounds. Matches between standards list and libraries with identical name are first discovered and removed. Among the remaining set of compounds, any compounds that have the same molecular formula or monoisotopic mass are examined. If compounds are structurally identical and differ only trivially in their name, the compounds the compound in the standards data is renamed to the form that exists in the library.

# Process Data

```
# to run this script, set the working directory to the directory containing this file
# e.g., setwd("~/S4_Metabolomics_Analysis_Script/")

# [1] Import Standards
standards <- readr::read_tsv("standards/standards_list_final.tsv")
#> 
#> ── Column specification ────────────────────────────────────────────────────────
#> cols(
#>   compoundName = col_character(),
#>   sample_num_1 = col_double(),
#>   sample_num_2 = col_double()
#> )

# [2] Water Results
# water results are assessed based on quant profile

# water top match
neg_water_top_match_results <- get_experiment_results("mzrolldb/Neg_water.mzrollDB", standards, NULL, correct_top_match_by_quant)
pos_water_top_match_results <- get_experiment_results("mzrolldb/Pos_water.mzrollDB", standards, NULL, correct_top_match_by_quant)
water_top_match_results <- combine_modes(neg_water_top_match_results, pos_water_top_match_results)

# water enrichment
neg_water_enrichment_results <- get_experiment_results("mzrolldb/Neg_water.mzrollDB", standards, NULL, correct_enrichment_by_quant)
pos_water_enrichment_results <- get_experiment_results("mzrolldb/Pos_water.mzrollDB", standards, NULL, correct_enrichment_by_quant)
water_enrichment_results <- combine_modes(neg_water_enrichment_results, pos_water_enrichment_results)

#[3] RT Table
# build an rt_table based on quant profile results
rt_table <- get_rt_table(water_top_match_results)
neg_rt_table <- rt_table %>% dplyr::filter(mode == "neg")
pos_rt_table <- rt_table %>% dplyr::filter(mode == "pos")

# [4] Yeast Results
# score group results based on RT results from water samples

# yeast enrichment
neg_yeast_enrichment_results <- get_experiment_results("mzrolldb/Neg_yeast.mzrollDB", standards, neg_rt_table, correct_enrichment_by_rt)
pos_yeast_enrichment_results <- get_experiment_results("mzrolldb/Pos_yeast.mzrollDB", standards, pos_rt_table, correct_enrichment_by_rt)
yeast_enrichment_results <- combine_modes(neg_yeast_enrichment_results, pos_yeast_enrichment_results)

# yeast top match 
neg_yeast_top_match_results <- get_experiment_results("mzrolldb/Neg_yeast.mzrollDB", standards, neg_rt_table, correct_top_match_by_rt)
pos_yeast_top_match_results <- get_experiment_results("mzrolldb/Pos_yeast.mzrollDB", standards, pos_rt_table, correct_top_match_by_rt)
yeast_top_match_results <- combine_modes(neg_yeast_top_match_results, pos_yeast_top_match_results)

# [5] TPR Results
scores <- seq(0, 150, 3)

tpr_results_water_enrichment <- get_tpr_results(water_enrichment_results, scores) %>%
  dplyr::mutate(dataset="water enrichment", background="water", type="enrichment")

tpr_results_water_top_match <- get_tpr_results(water_top_match_results, scores) %>%
  dplyr::mutate(dataset="water top match", background="water", type="top match")

tpr_results_yeast_enrichment <- get_tpr_results(yeast_enrichment_results, scores) %>%
  dplyr::mutate(dataset="yeast enrichment", background="yeast", type="enrichment")

tpr_results_yeast_top_match <- get_tpr_results(yeast_top_match_results, scores) %>%
  dplyr::mutate(dataset="yeast top match", background="yeast", type="top match")

tpr_results <- rbind(tpr_results_water_top_match, tpr_results_water_enrichment, tpr_results_yeast_top_match, tpr_results_yeast_enrichment)
```

# Plot results

## Water Enrichment Plot

```
ggplot(
  tpr_results_water_enrichment,
  aes(threshold, TPR)) +
  geom_point(size=5) +
  geom_smooth(span=2,linetype = "dashed") +
  ggtitle("Precision Curve Water Spike") +
  xlab("Hypergeometic Score Threshold") +
  theme_bw(base_size = 14) +
  scale_colour_brewer(palette = "Set1")
#> `geom_smooth()` using method = 'loess' and formula 'y ~ x'
```

## Water Top Match Plot

```
ggplot(
  tpr_results_water_top_match,
  aes(threshold, TPR)) +
  geom_point(size=5) +
  geom_smooth(span=2,linetype = "dashed") +
  ggtitle("Precision Curve Water Spike") +
  xlab("Hypergeometic Score Threshold") +
  theme_bw(base_size = 14) +
  scale_colour_brewer(palette = "Set1")
#> `geom_smooth()` using method = 'loess' and formula 'y ~ x'
```

## Yeast Enrichment Plot

```
ggplot(
  tpr_results_yeast_enrichment,
  aes(threshold, TPR)) +
  geom_point(size=5) +
  geom_smooth(span=2,linetype = "dashed") +
  ggtitle("Precision Curve Water Spike") +
  xlab("Hypergeometic Score Threshold") +
  theme_bw(base_size = 14) +
  scale_colour_brewer(palette = "Set1")
#> `geom_smooth()` using method = 'loess' and formula 'y ~ x'
```

## Yeast Top Match Plot

```
ggplot(
  tpr_results_yeast_top_match,
  aes(threshold, TPR)) +
  geom_point(size=5) +
  geom_smooth(span=2,linetype = "dashed") +
  ggtitle("Precision Curve Water Spike") +
  xlab("Hypergeometic Score Threshold") +
  theme_bw(base_size = 14) +
  scale_colour_brewer(palette = "Set1")
#> `geom_smooth()` using method = 'loess' and formula 'y ~ x'
```

## Water Plot

```
ggplot(
  tpr_results %>% dplyr::filter(background =="water"),
  aes(threshold, TPR, color=dataset)) +
  geom_point(size=5) +
  geom_smooth(span=2,linetype = "dashed") +
  ggtitle("Precision Curve Water Spike") +
  xlab("Hypergeometic Score Threshold") +
  theme_bw(base_size = 14) +
  scale_colour_brewer(palette = "Set1")
#> `geom_smooth()` using method = 'loess' and formula 'y ~ x'
```

## Yeast Plot

```
ggplot(
  tpr_results %>% dplyr::filter(background =="yeast"),
  aes(threshold, TPR, color=dataset)) +
  geom_point(size=5) +
  geom_smooth(span=2,linetype = "dashed") +
  ggtitle("Precision Curve Water Spike") +
  xlab("Hypergeometic Score Threshold") +
  theme_bw(base_size = 14) +
  scale_colour_brewer(palette = "Set1")
#> `geom_smooth()` using method = 'loess' and formula 'y ~ x'
```

## Single Combined Plot

```
tpr_results_renamed <- tpr_results %>%
  dplyr::rename(Dataset = dataset,
                `Total Positive Rate` = TPR)
ggplot(
  tpr_results_renamed,
  aes(threshold, `Total Positive Rate`, color=Dataset, shape=Dataset)) +
  geom_point(size=5) +
  geom_smooth(span=1,linetype = "dashed") +
  ggtitle("Precision Curve Water Spike") +
  xlab("Hypergeometic Score Threshold") +
  theme_bw(base_size = 14) +
  theme(legend.position=c(0.8,0.2), legend.background = element_rect(fill="white", size = 0.5, linetype="solid", colour="black")) +
  scale_colour_brewer(palette = "Set1")
#> `geom_smooth()` using method = 'loess' and formula 'y ~ x'
```

## Water - Yeast Subplots

```
ggplot(
  tpr_results,
  aes(threshold, TPR, color=dataset)) +
  geom_point(size=5) +
  geom_smooth(span=2,linetype = "dashed") +
  ggtitle("Precision Curve Water Spike") +
  xlab("Hypergeometic Score Threshold") +
  theme_bw(base_size = 14) +
  scale_colour_brewer(palette = "Set1") +
  facet_wrap(~background)
#> `geom_smooth()` using method = 'loess' and formula 'y ~ x'
```

## Enrichment - Top Match Subplots

```
ggplot(
  tpr_results,
  aes(threshold, TPR, color=dataset)) +
  geom_point(size=5) +
  geom_smooth(span=2,linetype = "dashed") +
  ggtitle("Precision Curve Water Spike") +
  xlab("Hypergeometic Score Threshold") +
  theme_bw(base_size = 14) +
  scale_colour_brewer(palette = "Set1") +
  facet_wrap(~type)
#> `geom_smooth()` using method = 'loess' and formula 'y ~ x'
```
